# Distribution, genetic diversity and potential spatiotemporal scale of alien gene flow in crop wild relatives of rice (*Oryza* spp.) in Colombia

**DOI:** 10.1186/s12284-017-0150-9

**Published:** 2017-04-18

**Authors:** Evert Thomas, Eduardo Tovar, Carolina Villafañe, José Leonardo Bocanegra, Rodrigo Moreno

**Affiliations:** 1 Bioversity International, Lima, Peru; 2 The Alexander von Humboldt Biological Resources Research Institute, Laboratory of Conservation Genetics, Bogota, Colombia; 3 Ministry of Environment and Sustainable Development, Genetic Resources Group, Bogota, Colombia; 4 The Alexander von Humboldt Biological Resources Research Institute, International Affairs, Policy and Cooperation Office, Bogota, Colombia

**Keywords:** Alien gene flow, Crop wild relatives, Genetic diversity, Habitat suitability, Rice cultivation, Colombia, Climate change

## Abstract

**Background:**

Crop wild relatives (CWRs) of rice hold important traits that can contribute to enhancing the ability of cultivated rice (*Oryza sativa* and *O. glaberrima*) to produce higher yields, cope with the effects of climate change, and resist attacks of pests and diseases, among others. However, the genetic resources of these species remain dramatically understudied, putting at risk their future availability from *in situ* and *ex situ* sources. Here we assess the distribution of genetic diversity of the four rice CWRs known to occur in Colombia (*O. glumaepatula*, *O. alta*, *O. grandiglumis*, and *O. latifolia*). Furthermore, we estimated the degree of overlap between areas with suitable habitat for cultivated and wild rice, both under current and predicted future climate conditions to assess the potential spatiotemporal scale of potential gene flow from GM rice to its CWRs.

**Results:**

Our findings suggest that part of the observed genetic diversity and structure, at least of the most exhaustively sampled species, may be explained by their glacial and post-glacial range dynamics. Furthermore, in assessing the expected impact of climate change and the potential spatiotemporal scale of gene flow between populations of CWRs and GM rice we find significant overlap between present and future suitable areas for cultivated rice and its four CWRs. Climate change is expected to have relatively limited negative effects on the rice CWRs, with three species showing opportunities to expand their distribution ranges in the future.

**Conclusions:**

Given (i) the sparse presence of CWR populations in protected areas (ii) the strong suitability overlap between cultivated rice and its four CWRs; and (iii) the complexity of managing and regulating areas to prevent alien gene flow, the first priority should be to establish representative *ex situ* collections for all CWR species, which currently do not exist. In the absence of studies under field conditions on the scale and extent of gene flow between cultivated rice and its Colombian CWRs, effective *in situ* conservation might best be achieved through tailor-made management plans and exclusion of GM rice cultivation in areas holding the most genetically diverse CWR populations. This may be combined with assisted migration of populations to suitable areas where rice is unlikely to be cultivated under current and future climate conditions.

**Electronic supplementary material:**

The online version of this article (doi:10.1186/s12284-017-0150-9) contains supplementary material, which is available to authorized users.

## Background

There is increasing global interest in crop wild relatives (CWRs) for the important contribution they can make to current endeavors to sustain and increase global food production (Vincent et al. 2013; Khoury et al. 2015; Castañeda-Álvarez et al. 2016). CWRs are wild plant taxons related to crops and their original progenitors, which have potential to contribute adaptive, nutritional, agronomic and other useful traits for crop improvement. In light of climate change, the need to improve the adaptive capacity of crop taxa for resisting acute and chronic biotic and abiotic stress, has never been more pressing. CWRs offer a treasure trove that can serve such capacity improvement (Atwell et al. 2014; Dempewolf et al. 2014). Many CWRs are adapted to conditions of environmental hardship, making them less susceptible to the effects of climate change, or even allowing them to prosper when climate gets hotter and dryer (Thomas et al. 2016). CWRs have permitted breeders to build in pest and disease resistance, abiotic stress tolerance, and quality traits in a growing number of food crops (Hajjar and Hodgkin 2007; Brar and Singh 2011). Over the past decades there has been a stable increase in the rate of release of cultivars containing genes from CWRs (Hajjar and Hodgkin 2007).

The *Oryza* genus is one of the most extensively studied genepools regarding the actual and potential use of CWR genes for crop improvement (Jena 2010; Brar and Singh 2011; Sanchez et al. 2013). The genus is widely distributed in tropical and sub-tropical regions around the globe (Atwell et al. 2014) and consists of two cultivated (*O. sativa* L. and *O. glaberrima* Steud.) and 22 wild species (Vaughan et al. 2003; Jena 2010). Four species complexes have been identified: the *O. sativa, O. officinalis, O. ridleyi*, and *O. meyeriana* complexes (Vaughan 1989; Vaughan et al. 2003)*.* Both cultivated species pertain to the AA genome *(O. sativa* complex) making gene transfer from wild relatives in the same complex relatively easy. However, many beneficial genes have also been successfully transferred to cultivated rice from the more distantly related species (Jena 2010; Brar and Singh 2011; Sanchez et al. 2013).

Central and South America are home to four rice CWR species: the diploid *O. glumaepatula* Steud (2n = 24, AA), included in the *O. sativa* complex, and the three allotetraploid species *O. alta* Swallen, *O. grandiglumis* (Doell) Prod., and *O. latifolia* Desv. included in the *O. officinalis* complex (2n = 48, CCDD) (Veasey et al. 2008a). All these species have either been identified as having high potential for use in cultivated rice improvement programs, or have already been the subject of actual gene transfer (Vaughan et al. 2003; Jena 2010; Brar and Singh 2011; Niroula et al. 2012; Sanchez et al. 2013; Pak et al. 2013). However the genetic diversity and structure, as well as the *in situ* conservation status of these species remains severely understudied, jeopardizing the conservation and future use of their genetic resources. To date most studies have focused on *O. glumaepatula* (Akimoto et al. 1998; Buso et al. 1998; Brondani et al. 2005; Karasawa et al. 2007a; Karasawa et al. 2007b; Veasey et al. 2008b; Karasawa et al. 2012), and much less on the other three species (Quesada et al. 2002; Arrieta-Espinoza et al. 2005; Veasey et al. 2008b; Veasey et al. 2011).

The distribution pattern of genetic diversity across a species´ range can be influenced by climate change, notably through alteration of geographical connectivity and associated modification of gene flow patterns within and across populations (Huang and Schaal 2012). Rapid changes in population size during past glacial cycles have been related to processes of strong genetic drift (Knowles and Richards 2005). When populations get isolated in areas with variable environmental conditions, for example as a consequence of range contraction, divergent selection pressures for different populations can lead to local adaptation (Chen et al. 2012). The last glaciation (22,000–13,000 years before present [BP]) is the most important period of climate change in recent history that had a profound impact on the genetic distribution of many species across the world, including *O. rufipogon*, the progenitor of cultivated Asian rice (Huang and Schaal 2012). Owing to lower air temperatures, lower precipitation levels and increased water stress associated with lower atmospheric CO_2_ concentrations, many species have experienced range contractions, leading to patterns of genetic differentiation that remain evident in their genetic profiles (Thomas et al. 2012; Galluzzi et al. 2015; Thomas et al. 2015). Here we assess the potential impact of past climate change on the genetic profiles of the Colombian rice CWRs.

One of the potential future threats to the *in situ* conservation of rice CWRs in Colombia may relate to introgression of alien genes. GM rice has been legally permitted in the country for use as food since 2008 (CERA 2012). Although its cultivation is currently not allowed, the potential future introduction of GM rice in areas where wild species occur may lead to a risk of introgression of GMO genes into the gene pools of wild populations with potential effects on their adaptive potential, population dynamics and ecological roles, among others (Chen et al. 2004; Garcia and Altieri 2005). Snow et al. (2005) argued that the environmental effects of the introgression of GMO genes in wild species are generally neutral or insignificant. In rice CWRs, high rates of self-pollination can be expected to result in low risk of alien gene transfer (Stewart et al. 2003). However, low risk is not zero risk and even at very low cross-pollination rates, the likelihood of a transgene being quickly dispersed through wild receptor populations increases when it grants a significant adaptive advantage in resistance to pests and diseases, higher tolerance to abiotic stresses (drought, salinity), or higher yields (Ellstrand 2003).

The purpose of this study was threefold. First, we investigated the genetic diversity and structure of the four rice CWRs in part of their natural distribution range in Colombia. Following other studies (Waltari et al. 2007; Thomas et al. 2012; Thomas et al. 2015; Thomas et al. 2017), we carried out suitability modeling at the last glacial maximum (LGM; ~21,000 years BP) and spatially overlaid the results with the outcomes of genetic sampling, to evaluate the potential impact of the last glacial period on the current distribution of genetic diversity in all four species. Second, we estimated the degree of overlap between areas with suitable habitat for cultivated and wild rice, both under current and predicted future climate conditions to assess the potential spatiotemporal scale of potential gene flow from GM rice to its CWRs. This also allowed us to evaluate the potential effect of climate change on the *in situ* persistence of the rice CWRs. Third and last, we discuss the implications of our findings for the design of tailor-made *in situ* and *ex situ* conservation strategies aimed at maintaining and enhancing the natural genetic variability of Colombian rice CWRs, and ensuring the availability of their genetic resources for future use.

## Results

### Genetic diversity and spatial structure

For the tetraploid species, highest allelic richness was observed in *O. latifolia* with 117 alleles and an average of 10.6 per locus, followed by *O. alta* and *O. grandiglumis* with 46 and 43 alleles at averages of 4.2 and 3.9 alleles per locus, respectively (Additional file 1: Table S1). The diploid species *O. glumaepatula,* yielded a total of 30 alleles with an average of 2.7 alleles per locus (Additional file 2: Table S2). The latter value is in agreement with a study conducted by Karasawa et al. (2007a) with SSR markers in *O. glumaepatula* populations from Brazil which reported values of 3.09. High values of expected heterozygosity (H_E_) were observed in the tetraploid species. Average H_E_ across all loci was 0.57 for *O. latifolia*, 0.43 for *O. grandiglumis,* and 0.40 for *O. alta*. The lowest H_E_ value was found for *O. glumaepatula* (0.21).

**Fig. 1:**
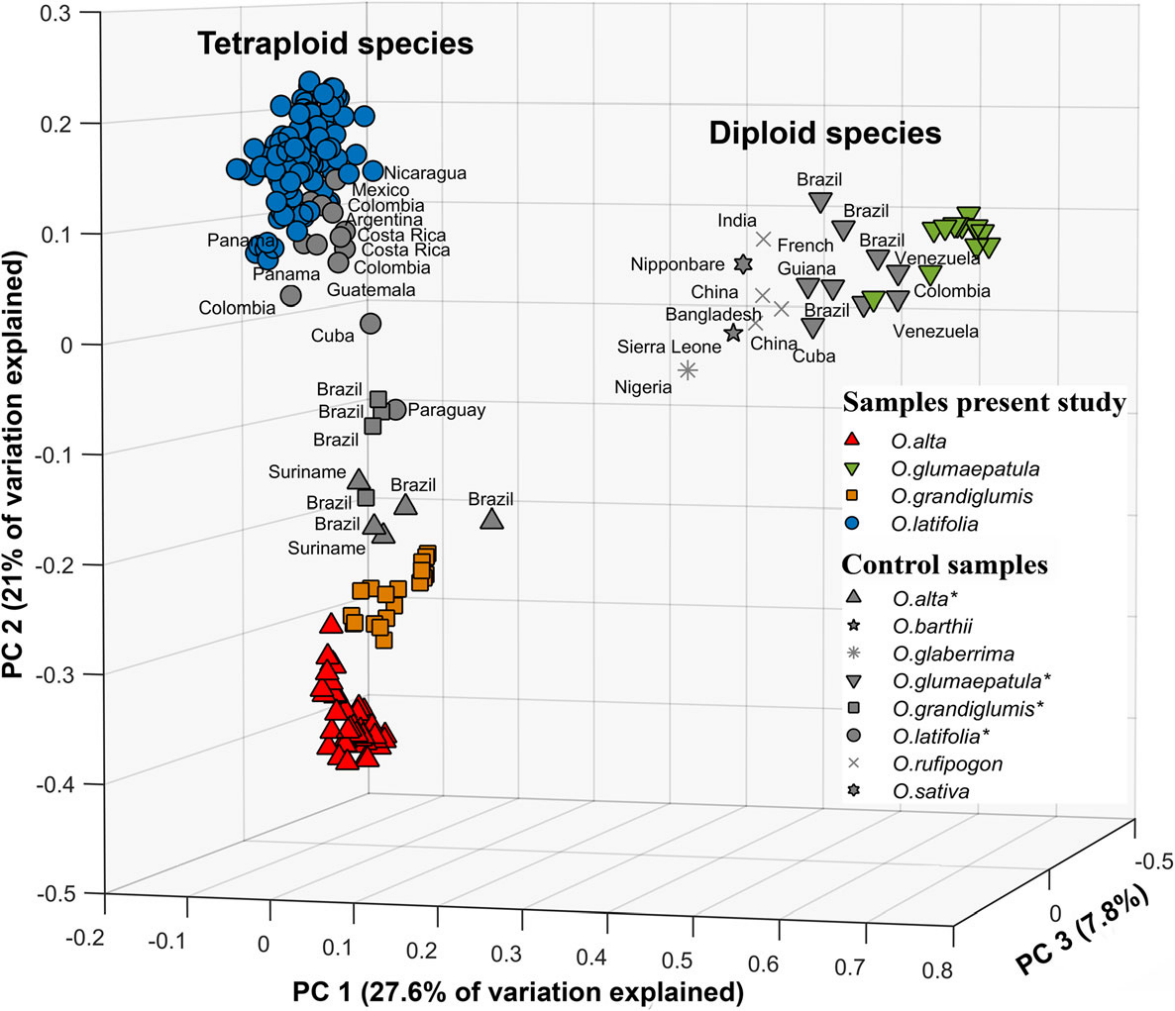
Principal coordinates analysis of all *Oryza* individuals sampled for the present study and reference samples, based on the Bruvo genetic distance

A scatter plot of the first two axes of a PCoA undertaken for all species samples (Fig. 1), shows a clear separation of the diploid and the tetraploid species groups, with some degree of overlap between species within groups. The partial overlap between species samples in Fig. 1 may be partly due to the limitations of the Bruvo genetic distance metric to clearly separate all the different species in three-dimensional ordination space. However, Fig. 1 does distinctively group the Colombian CWR samples according to their taxonomic affiliation and separates them from reference samples pertaining to the same species, confirming their divergent genetic make-up.

**Fig. 2:**
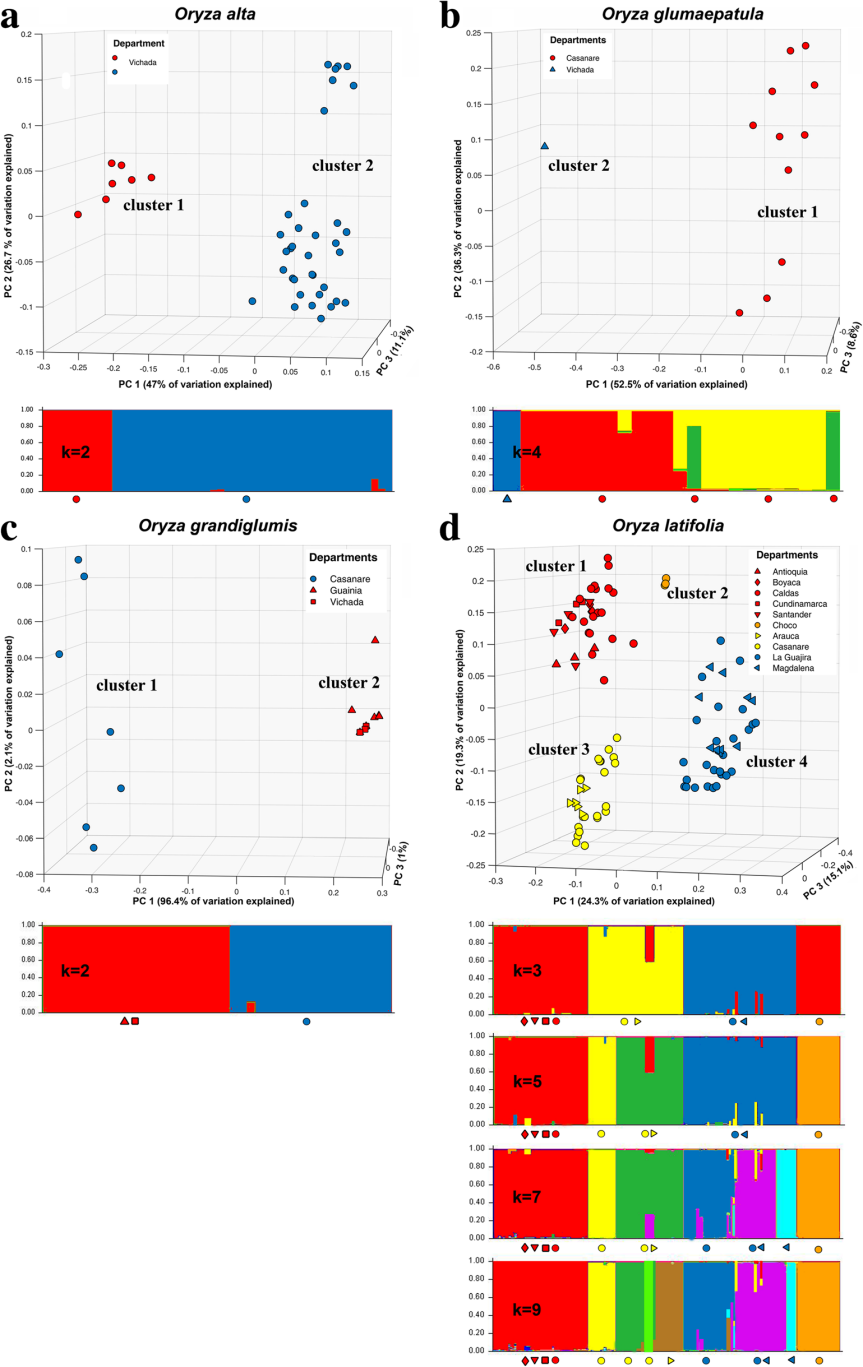
Results of species-specific principal coordinates analysis based on the Bruvo genetic distance and Bayesian admixture proportions identified by STRUCTURE (Pritchard et al. 2000) of individual plants of **a** *O. alta*, **b** *O. glumaepatula*, **c** *O. grandiglumis*, **d** *O. latifolia*. The genetic groups in the PCoA plots were identified through hierarchical cluster analysis (>95% branch support; see Additional file 3: Figure S1). *Symbols* and *colors* used in the PCoA plots are associated with STRUCTURE clusters for different values of K

**Fig. 3:**
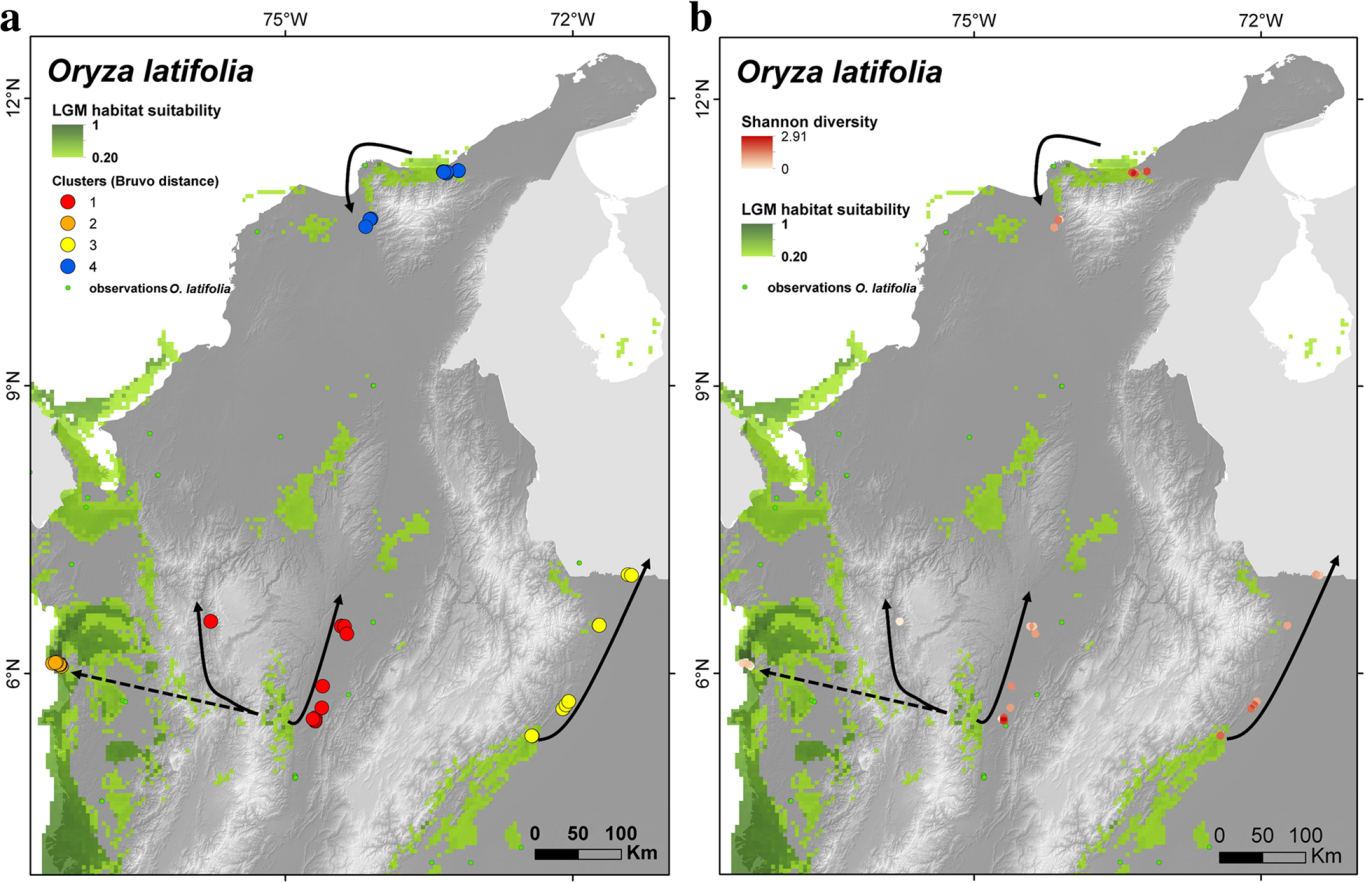
Combination of LGM suitability map of *Oryza latifolia* and (**a**) the geographical distribution of genetic groups (**b**) Shannon diversity index measured at sampling sites. The *arrows* indicate the potential direction of range expansion from putative glacial refugia. The *dotted arrow* suggests that range expansion is likely to be relatively recent, and possibly of anthropogenic origin

Species-specific PCoAs allowed visualization of the differentiation between the spatially coherent genetic groups identified by hierarchical cluster analysis in all species (Fig. 2 and Additional file 3: Figure S1). Bayesian cluster analysis yielded very similar results for *O. grandiglumis* (two clusters) and provided more detail on the small-scale genetic structure of *O. alta*, *O. glumaepatula* and *O. latifolia*. ΔK values are shown in Additional file 4: Figure S2. For *O. alta* (K = 3) two subclusters were identified in cluster 2, corresponding with the two isolated groups of points in the upper and lower parts in the PCoA diagram, respectively. However, these two subgroups were not geographically coherent. Results of ΔK computation for *O. glumaepatula* showed support for K = 2 and 4. For K = 2 (results not shown) part of the individuals from Casanare department (cluster 1 in Fig. 2b and Additional file 4: Figure S2) were grouped together with cluster 2 in spite of their spatial isolation. For K = 4 the individuals of cluster 2 were assigned to a separate group, while three subgroups were identified among the individuals from cluster 1, even though these were separated by less than 3 km as the crow flies. Highest cluster richness was found for *O. latifolia* that was also the most extensively sampled species. Hierarchical cluster analysis identified four main clusters largely according to the geographical locations of sampled individuals (Fig. 3). However, Fig. 2d suggests a high degree of affinity between clusters 1 and 2. Results of ΔK computation for *O. latifolia* showed support for K = 5 and 7 (Additional file 4: Figure S2). The most parsimonious scenario (K = 5), grouped the individuals in the same clusters as the hierarchical cluster analysis, but identified two subgroups in cluster 3. For K = 7, cluster 4 was further separated in three subclusters. Overall, the large majority of individuals were consistently assigned to discrete genetic groups with little evidence of admixture within clusters. Furthermore, the subgroups identified in clusters 3 and 4 were arranged sequentially along the more or less linear gradients shown in Fig. 3.

All main clusters identified in the PCoA plots were separated by large geographical distances in *O. latifolia, O. glumaepatula* and *O. grandiglumis*, and by the Vita River, one of the tributaries of the Orinoco River, in *O. alta* (Fig. 3, Additional file 5: Figure S3, Additional file 6: Figure S4, and Additional file 7: Figure S5). These barriers can be expected to have constrained gene flow between the different groups, and hence are likely to have contributed to their genetic differentiation. This is corroborated by the significant isolation by distance correlations we found for all four species when considering all individuals sampled (*p* < 0.0001 in all cases; Additional file 8: Table S3). Significant correlations were also found at genetic cluster level, except for cluster 2 in *O. latifolia*, which was expected as plant individuals from this clusters where collected at very short distances (Additional file 8: Table S3).

Genetic distance between genetic groups as measured by G_ST_ (Nei 1973) was 0.129, 0.303 and 0.655 for the two clusters identified in *O. alta*, *O. grandiglumis* and *O. glumaepatula*, respectively, and varied between 0.068 and 0.184 for the four clusters identified in *O. latifolia*.

### LGM habitat suitability and relation with genetic diversity distribution

To explore how the last glacial period may have contributed to structuring the distribution of genetic diversity of the wild relatives, we overlaid maps of their genetic diversity and LGM habitat suitability. For all species suitable habitat conditions seemed to have prevailed in the vicinities of the identified genetic clusters (Fig. 3, Additional file 5: Figure S3, Additional file 6: Figure S4, and Additional file 7: Figure S5), which could imply that the origin of these clusters may partly be due to genetic differentiation in isolated refugia during the last glacial period. This pattern is most apparent for *O. latifolia*. Combined interpretation of the distribution of genetic groups (Fig. 3a; beta diversity) and diversity scores at individual sampling sites of this species (Fig. 3b; alpha diversity) can help unraveling potential glacial and post-glacial range dynamics.

Figure 3b shows that the highest levels of genetic diversity observed in clusters 1, 2 and 3 are geographically closest to areas which may have been suitable during the LGM, with a trend of decreasing levels of genetic diversity in populations when moving away from these areas, in line with observed isolation-by-distance patterns (Additional file 8: Table S3). This could suggest that genetic diversity may have been concentrated in these suitable areas during the last glacial period, leading to genetic differentiation of the different groups, and that range expansion during the warming Holocene may have occurred along the gradient of decreasing diversity, as indicated by the arrows in Fig. 3. The limited genetic differentiation between the cluster from the Pacific coast and the central Colombian valleys (clusters 2 and 1, respectively), evident from the PCoA scatter plot (Fig. 2d), suggests that the former may represent a recent introduction, possibly even of anthropogenic nature. If correct, it is possible that although habitat conditions may have been suitable in the Pacific coast area, the species was not (yet) present there; otherwise one would expect higher diversity scores.

### Current and future habitat suitability of cultivated rice and its wild relatives

**Fig. 4:**
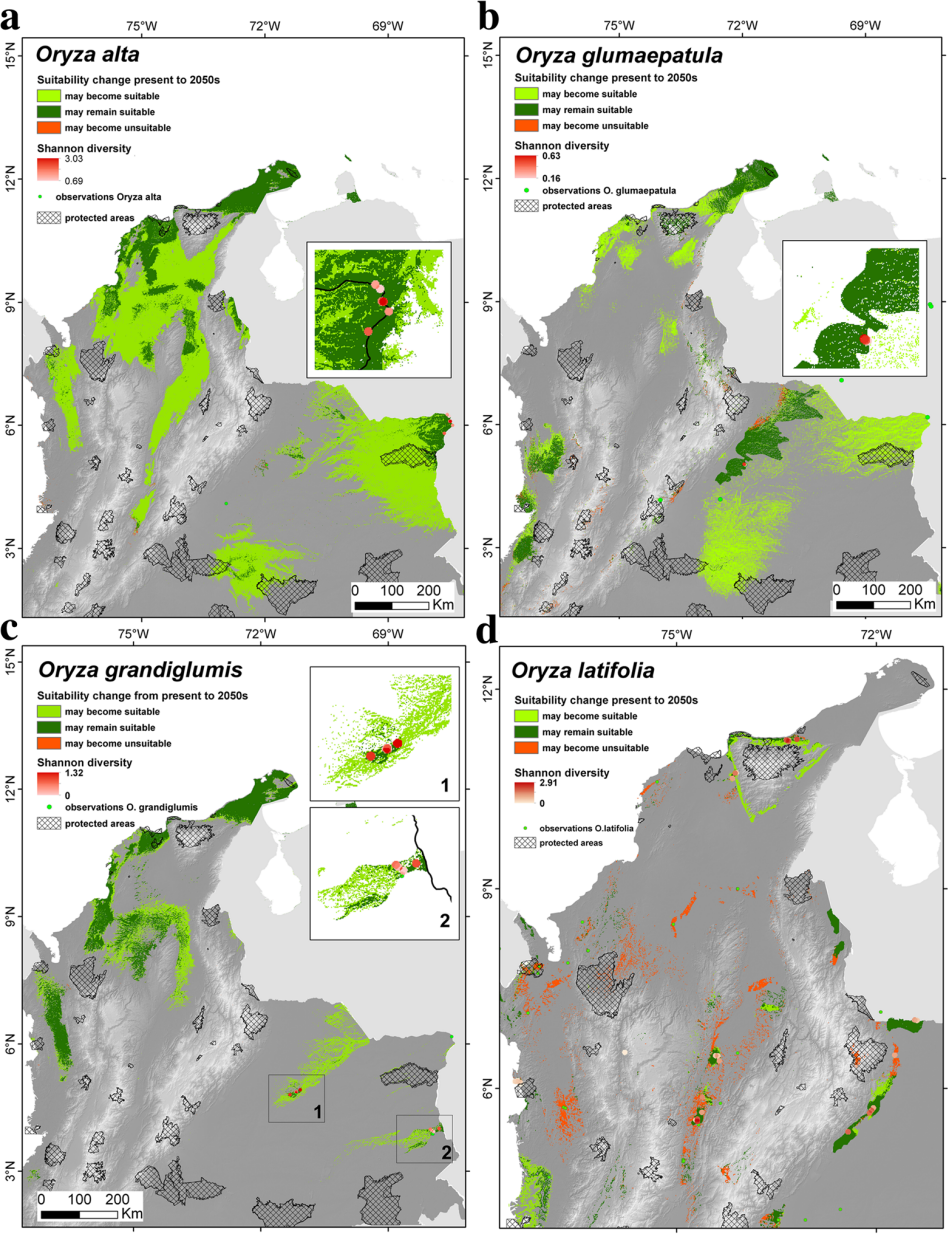
Predicted changes in habitat suitability from present to the 2050s of **a** *O. alta,* **b** *O. glumaepatula*, **c** *O. grandiglumis*, and **d** *O. latifolia*, compared with Shannon diversity index at sampling sites

A comparison of habitat suitability under current and future climate conditions suggests that all CWRs except *O. latifolia* might be able to expand their distribution ranges in the mid-term future (Fig. 4), implying that their genetic resources would not be seriously threatened by the effects of climate changes on their range sizes. For *O. latifolia* a slightly less optimistic result was obtained, with most areas currently identified as suitable but likely to become unsuitable in the future, and relatively limited opportunities for future suitability gains. In spite of this, nearly all areas where we observed the highest levels of genetic diversity are expected to remain suitable in the future. In only judging areas that are identified by more than half of the 30 ‘suitable’ future model projections, it is important to note that we may be overestimating actual climate impacts, since much broader distributions of suitable habitat were obtained when using lower thresholds (not shown).

**Fig. 5:**
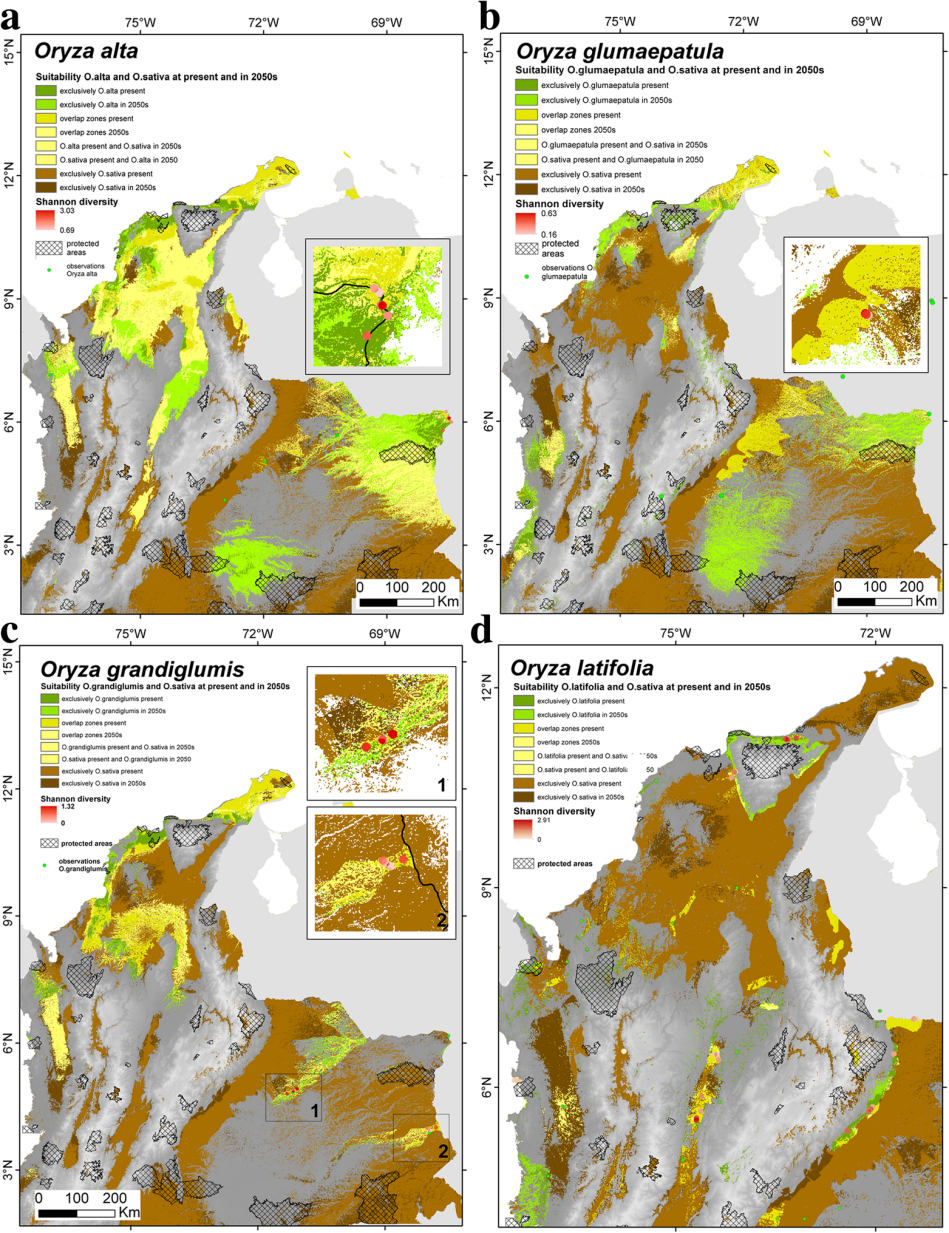
Distribution and overlap of suitable areas of cultivated rice *O. sativa* (irrigated and rainfed) and its wild relatives **a** *O. alta*, **b** *O. glumaepatula*, **c** *O. grandiglumis* and **d** *O. latifolia* under current and expected future (2050s) climate conditions. Please note that overlap zones of suitable areas of cultivated rice and its wild relatives whereby habitat suitability of at least one of both is expected to occur under predicted future climate conditions (three different possible combinations) are all highlighted in *pale yellow*

Our modelling exercise has identified extensive suitable areas for rice cultivation (Additional file 9: Figure S6). Climate change impact is expected to be more severe for rainfed rice than for irrigated rice, (based on the assumption of unrestricted availability of irrigation water, which is unrealistic in many areas, such as the Guajira peninsula), while the overall impact on cultivated rice (rainfed and irrigation rice) may be most serious in the eastern Llanos areas and the northern part of the country (Additional file 9: Figure S6). Most of the areas where the different CWR species are currently known to occur, or might occur, based on suitable habitat conditions, overlap with areas where rice cultivation is already ongoing, or likely to be possible either under either current or future climate conditions (Fig. 5). Additionally, a combination of the distribution of field observations and the results of our modeling exercise suggest an extremely poor coverage of CWRs in protected areas. This implies that in most of the areas where these species occur or are likely to occur GM rice could potentially be cultivated.

## Discussion

### Genetic diversity and spatial structure

Results suggest that the Colombian populations of rice CWRs we studied hold relatively high levels of unique diversity compared to samples (Fig. 1) and studies (see below) from other parts in their distribution ranges. Differences in allelic richness found for the four species partly reflect sampling intensity, but higher values observed for the allotetraploid species are also very likely partly due to the higher probability of encountering different alleles per locus compared to diploid species (maximum of four versus two per locus, respectively). The chance of observing a (partially) heterozygous locus is also greater in allotetraploids than in diploids for the same reason.

Compared to other studies (Additional file 10: Table S4), the H_O_ and H_E_ values (0.14 and 0.21, respectively) we found for *O. glumaepatula* could be considered relatively high and the inbreeding coefficient relatively low (0.37), when taking into account the small number of samples studied (*n* = 25). Karasawa et al. (2007b) found H_O_ and H_E_ to range from 0.10 to 0.23 and from 0.37 to 0.57, respectively, in populations of 100 individuals each along a latitudinal gradient in Brazil, genotyped with SSR markers. The fixation index varied between 0.37 and 0.82. In another SSR study of 414 individuals from Brazil, Brondani et al. (2005) found values for Ho and H_E_ at the low end of the ranges found by Karasawa et al. (2007b) (0.03 and 0.12, respectively). They accordingly found a high inbreeding coefficient (F_IS_ = 0.79), indicating a predominantly autogamous reproduction in their study populations. Similarly, isoenzyme marker studies –known to result in lower H_E_ scores than with SSRs (Gao et al. 2002)– from Brazil revealed low H_E_ values (0.04 and 0.06) and high F_IS_ values (>0.9) in a large number of individuals (333 and 1090) (Akimoto et al. 1998; Veasey et al. 2008a), respectively), pointing to a predominantly autogamous reproduction.

Karasawa et al. (2007b) argued that the reproductive system of *O. glumaepatula* may be variable both among and within populations, ranging from complete selfing to a mixed system with predominance of selfing, which could explain the variable observations of the inbreeding coefficient across populations. If correct, the Colombian populations we sampled may have a mixed mating system, characterized by a low but significant degree of outcrossing, evidenced by the relatively low inbreeding-coefficient compared to literature (Additional file 10: Table S4). One possibility that merits further research is whether the mating system of *O. glumaepatula* may be influenced by the ecological conditions of its habitat. Karasawa et al. (2007b) found the lowest values of inbreeding in a population from the Brazilian savanna, the same ecosystem where also the Colombian samples were collected. Possibly more open vegetation (savanna) may stimulate mixed mating systems while selfing may be an ecological response to more closed vegetation types (rainforest).

To our knowledge, diversity studies based on SSR markers are non-existent for the *Oryza* tetraploid species, complicating comparisons with other studies which are mainly based on isoenzymes (Karasawa et al. 2012). However, high values of H_E_ observed in the three species (0.40–0.57) suggest the Colombian populations hold high genetic diversity. For example, Sun et al. (1998) found a H_E_ value of *only* 0.25 across ten populations of *Elymus fibrosus*, a wild Poaceae species. Previous studies, based on different molecular techniques have repeatedly observed a genetic relatedness between *O. alta* and *O. grandiglumis* (Wang et al. 1992; Federici et al. 2002; Bao and Ge 2003 using Restriction Fragment Length Polymorphism; Aggarwal et al. 1997; Aggarwal et al. 1999 using total DNA hybridization and Amplified Fragment Length Polymorphism; and Joshi et al. 2000 using Inter Simple Sequence Repeats). The Colombian populations we studied did show some affiliation, but could nonetheless clearly be separated from each other in ordination space. Overall, our findings confirm the clear genetic differences in species pertaining to the CCDD genome observed by Aggarwal et al. (Aggarwal et al. 1996) and supported by others (Ge et al. 1999; Zamora et al. 2003; Veasey et al. 2008a).

### LGM habitat suitability and relation with genetic diversity distribution

As suggested by the relatively elevated G_ST_ values we found (Hedrick 2005; Jost 2008; Meirmans and Hedrick 2011), genetic groups within all four species showed high degrees of genetic differentiation. All the genetic groups could be linked to areas that are likely to have remained suitable for the respective species in the late Pleistocene. This could suggest that they genetically differentiated as a consequence of prolonged isolation of their source populations owing to range contractions that took place in response to the drying and cooling effects of the last glacial period. Similar evidence exists for the genetic differentiation of *O. rufipogon* in Southeast Asia (Fuller et al. 2010; Huang and Schaal 2012). Here this trend was most apparent for three of the four main genetic groups distinguished in *O. latifolia*. For each of these groups the probable directions of range expansion from the putative glacial refugia in which they are likely to have differentiated could be inferred from decreasing diversity trends when moving away from the putative refugia. Similar findings have been documented for other neotropical species (Thomas et al. 2012; Thomas et al. 2015).

Within clusters, gene flow seemed to occur at relatively short distances in all species, based on our finding of high G_ST_ values and strong isolation by distance patterns (cf. Schaal et al. 1998) in all but one of the clusters, even those with a relatively narrow geographic distribution (notably *O. alta* and *O. grandiglumis*). These observations are in line with the findings of (Veasey et al. 2011) and may be due to the reduced probability of long-distance pollination events resulting from the combination of the species´ predominantly autogamous reproduction systems and short gene flow distances expected from findings for other species in the *Oryza* genus (see below). Also the subgroups detected by Bayesian cluster analysis for clusters 3 and 4 of *O. latifolia* (Fig. 2d) might be explained by the same gene flow dynamics whereby sequential populations along the almost linear gradients of range expansion have been engaging in processes of genetic differentiation ever since their establishment. Further research is necessary to either confirm or refute this hypothesis.

### Potential current and future niche overlap and risk of alien gene transfer

Suitability modeling under current and future climate conditions suggests that there is a strong spatiotemporal overlap between suitable areas of cultivated rice (whether rainfed or irrigated), and its four CWRs. Although niche suitability is not necessarily followed by cultivation practice, this outcome is indicative of the potential scale of gene flow between GM rice and its CWRs. Measures of gene flow frequencies are lower than 1% between cultivated rice and below 3% between cultivated and wild rice (Chen et al. 2004). The chance of actual transfer of GM genes to wild populations is highest for *O. glumaepatula* which has an AA genome type and hence is genetically compatible with *O. sativa* (Naredo et al. 1997; Naredo et al. 1998). Hybridization between *O. sativa* and other *Oryza* AA genome types is feasible and has been evidenced in several studies (Song et al. 2003; Chen et al. 2004; Wang et al. 2006), including *O. glumaepatula* (Jena 2010). However, it does not seem to occur very frequently under natural conditions. Usually, this type of interspecific hybridization depends on an extensive embryo rescue and retro-crossing efforts in order to obtain fertile hybrids (Vaughan et al. 2003). Gene flow between cultivated rice and the tetraploid CWRs is even less probable. Crosses between AA and CCDD genomes are feasible (Brar and Singh 2011), but are very difficult to obtain, even under artificial conditions (Mathias Lorieux, pers. comm.).

In addition to genetic compatibility issues, some additional barriers to gene flow prevail. Importantly, pollination in cultivated rice is mainly autogamous, due to flower morphology and the short-term viability of pollen (<30 min) (Oka 1988; Song et al. 2001), thereby reducing the probability of gene flow and hybridization events. Furthermore, the bulk of cross-pollination between adjacent plants or fields of cultivated rice usually does not seem to exceed a few meters (at the 1.0% threshold (Messeguer et al. 2001; Rong et al. 2004; Rong et al. 2005)), although under certain circumstances pollen-mediated gene flow might potentially occur at distances of tens to hundreds of meters (Song et al. 2002; Song et al. 2003; Yao et al. 2008; Kanya et al. 2009). Consequently, spatial buffer zones ranging from a few meters to more than 250 m wide have been proposed to avoid alien gene flow from genetically modified to conventional rice or its wild relatives (Rong et al. 2007; Kanya et al. 2009). While theoretically feasible for cultivated rice, this is much more difficult for rice CWRs, for which dispersal is conditioned by natural processes. Wild species can appear spontaneously along the margins, or even inside GM rice fields, and hence be exposed to alien gene flow, largely beyond human control.

Effective gene flow between GM rice and wild species is scarcely documented, possibly owing to the fact that GM rice is not yet commercially cultivated worldwide. The introgression of alien alleles in the sink population genome will ultimately depend on the manifestation, or not, of adaptive advantages. Alleles that grant some evolutionary advantage (reproduction and survival) may overcome almost any reproductive barrier, and may eventually become fixed in all exposed gene pools (Piálek and Barton 1997; Morjan and Rieseberg 2004). On the other hand, if an allele or gene does not improve the reproduction or survival of the plant, the probability that they get fixed is very low, and if they do succeed they will most likely remain as rare alleles. While the introgression of GM genes in rice CWRs might at best result in a competitive advantage for the respective sink populations, the impact on the ecological system they are part of may be less favorable and in the worst case lead to cascading effects. To date, few studies have been conducted on the scale, dynamics and potential detrimental environmental effects of gene flow between GM rice and its wild relatives under actual field conditions. Further research is clearly needed to properly assess or anticipate possible risks of alien gene flow in areas where GM rice and its CWRs overlap as is highlighted by the recent findings of Pu et al. (2014). In a large scale study across China, they found that several hundreds of insect species engage in pollen-mediated gene flow, some of which, like the European honey bee *Apis mellifera*, carry pollen at least 500 m from the pollen source. Furthermore, they showed that *A. mellifera*-mediated transgene flow from genetically modified to conventional varieties of cultivated rice was much higher than when the bees did not participate in gene flow.

Future research on gene transfer between GM rice and rice CWR in Colombia and beyond should therefore focus on identifying potential insect pollinators, and understanding CWRs’ phenology, gene flow distances, seeds and pollen longevity, and the adaptive advantage of any transgenic traits introduced. For example, our hypothesis that the *O. glumaepatula* populations we sampled may have a mixed mating system characterized by a significant degree of outcrossing (implying that these populations may be more susceptible to introgression of alien genes), requires exhaustive field testing to allow developing adequate *in situ* conservation strategies. In the absence of detailed knowledge on the actual probabilities of gene flow between GM rice and its wild relatives, including the tetraploid ones, a precautionary approach may be appropriate.

### *In situ* and *ex situ* conservation

The potential uses of CWR genes for improvement of cultivated rice is well documented and include: elongation ability, source of cytoplasmic male sterility, tolerance to heat (*O. glumaepatula*); resistance to brown plant hopper and bacterial blight (*O. latifolia*); resistance to striped stem-borer (*O. alta*); and high biomass production (*O. alta, O. latifolia, O. grandiglumis*) (Jena 2010; Brar and Singh 2011; Sanchez et al. 2013). Our finding that all species, except *O. latifolia*, are expected to predominantly experience range expansion and almost no contraction under climate change (Fig. 4), may indicate they contain useful genes permitting this adaptedness and plasticity (e.g. heat tolerance in *O. glumaepatula*), which may in turn have potential for introgression in cultivated rice, for which less optimistic results were obtained (Additional file 6*: Figure S6). Similar results were obtained in a study of ten Mesoamerican crop genepools and their CWRs (Thomas et al. 2016).

The use of CWR genes for rice improvement is contingent on the effective conservation and availability of their genetic resources, either through the conservation of viable populations under *in situ* conditions, or through the establishment and maintenance of *ex situ* collections. The fact that nearly all of the areas where the rice CWRs are known to occur in Colombia are located outside of protected areas and are also suitable for (GM) rice cultivation, complicates the possibility of *in situ* conservation in areas exempt from risks of alien gene flow. The current *ex situ* conservation status of Colombian rice CWR is similarly problematic. In spite of their high breeding potential and distinctive genetic makeup, Colombian rice CWRs are very poorly conserved in international and national gene banks. At the time of this research, the International Rice Research Institute (IRRI), held only two *O. latifolia* and one *O. glumaepatula* accessions from Colombia, while the International Center for Tropical Agriculture (CIAT) whose international gene bank is located in Cali, Colombia, conserved none. To our knowledge, national gene bank collections for any of the four CWRs do not exist in Colombia.

In light of the strong overlap between present and future suitable areas of cultivated rice and its four CWRs, the poor coverage of populations in protected areas, and the complexity of managing and regulating areas which are exempted from the possibility of alien gene flow (e.g. through a ban on cultivation of GM rice), a first priority should be to establish representative *ex situ* collections for all wild species. Priority populations of rice CWRs for the collection of accessions for *ex situ* conservation and the development of *in situ* conservation strategies are a combination of populations that hold the highest levels of genetic diversity (alpha diversity) and are complementary in terms of adaptive traits and their degree of genetic differentiation (beta diversity). In practical terms this would mean *in situ* conservation strategies should focus on those areas holding the highest genetic diversity for each of the genetic clusters. Additionally, the feasibility of establishing valuable populations, through assisted migration or assisted colonization, in areas holding favorable habitat conditions under current and/or future habitat for CWR, but not cultivated rice could be explored. According to our models such areas are likely to exist for all species.

However, it is clear that our genetic characterization data are not complete and other highly diverse and genetically differentiated populations may exist that were not included in our sample but nonetheless deserve attention for conservation. Based on the results obtained here, identification of areas where CWRs are either known or likely to occur (through botanical collections and suitability mapping, respectively) and that overlap with, or are spatially close to, areas that may have held suitable habitat conditions during the LGM, could, in the absence of genetic characterization data, be a useful first step to prioritize areas deserving further attention. Areas that have acted as glacial refugia are likely to contain higher levels of unique genetic diversity compared to expansion areas within species’ ranges (Thomas et al. 2012; Thomas et al. 2015; Thomas et al. 2016). While this hypothesis is corroborated most notably by our observations for *O. latifolia*, for all species there are multiple areas that correspond to these characteristics and hence should be targeted for future collection and characterization missions. Logically, the development and implementation of in situ conservation plans should be preceded by genetic (and phenotypic) characterization and the collection of representative germplasm samples for ex situ conservation. This will allow establishing a baseline of the genetic diversity and structure of the CWR populations under consideration to which observations from future monitoring efforts can be compared.

## Methods

### Plant materials

Between 2009 and 2012, leaf tissue of a total of 291 individuals was collected across the four Colombian rice CWRs: 175 belonged to *O. latifolia*, 41 to *O. grandiglumis*, 50 to *O. alta*, and 25 to *O. glumaepatula*. Field sampling was guided by suitability maps based on occurrence data obtained from botanical collection records. No collection permits were required since the Alexander von Humboldt Biological Resources Research Institute, ascribed to the Ministry of Environment and Sustainable Development, is exempted from this requirement (Presidential Decree 302 of 2003). Taxonomic identification of the different species was carried out based on a combination of morphological and molecular methods. The ploidy of the species was identified through RFLP (Restriction Fragment Length Polymorphism) analysis and comparison with reference samples of known taxonomic classification, grown from seeds obtained from the International Rice Research Institute (IRRI). Identification based on morphological traits was based on Vaughan (Vaughan 1989; Vaughan 2003) and discussed in Villafañe et al. (2007). Photographs of morphological traits distinguishing the species with CCDD genome are presented in Additional file 11: Figure S7.

To allow evaluating the genetic differentiation of Colombian samples compared with accessions from the same and other species collected elsewhere in the world, we included 40 reference samples: 23 from the *O. officinalis* complex comprising the allotetraploid species *O. alta, O. grandiglumis* and *O. latifolia*, and 17 from the *O. sativa* complex with representatives from the diploid species *O. rufipogon, O. sativa, O. bartthii, O. glaberrima*, and *O. glumaepatula* (Fig. 1; Additional file 12: Table S5).

### DNA extraction and microsatellite markers

DNA extraction was carried out from foliar material using the DNeasy Plant Mini Kit (QIAGEN). Total DNA quality was verified through visualization by horizontal electrophoresis in 0.8% w/v agarose gels stained with SYBR Safe (Invitrogen). DNA concentration was determined through quantification using the BioPhotometer spectrophotometer (Eppendorf).

Molecular characterization of the samples was undertaken with 11 previously evaluated Simple Sequence Repeats (SSR) markers (McCouch et al. 2002) selected from the rice database (http://www.gramene.org/markers/microsat) (Additional file 13: Table S6). A recent meta-analysis of 127 species (Mittell et al. 2015) has shown that this marker number is sufficient for characterizing a population's molecular genetic variation at comparable markers. Polymerase chain reactions (PCR) were carried out in a PTC-100TM thermal cycler (MJ Research) and consisted of a first cycle of denaturation at 94 °C for 3 min; followed by 30 cycles of 94 °C for 30 s, 55 °C for 30 s, and 72 °C for 1 min; and a final extension cycle at 72 °C for 5 min. The reaction mixture with a final volume of 50 μl had 100 ng of DNA template, 1× buffer (Tris-HCl 100 mM, KCl 500 mM), 3.0 mM of MgCl_2_, 0.2 mM of each dNTP, 0.3 μM of each primer, and 1 U of *Taq* polymerase. PCR products were visualized in 6% w/v polyacrylamide gels, 7.5 M of urea, stained with silver nitrate (AgNO_3_) (Bassam et al. 1991). Allele sizes were determined with the Quantity One software version 4.6.3 (Bio-Rad) through comparison with the 10 bp standard weight marker (Invitrogen™).

### Suitability modeling

We characterized the spatial distribution of favorable habitat conditions of cultivated rice and its four CWRs in Colombia, under different climatic conditions, by means of suitability mapping based on ensembles of modeling algorithms, implemented in the R package *BiodiversityR* (Kindt and Coe 2005). Habitat suitability of the CWRs during the LGM was modeled to obtain insights of the potential impact of the last glacial period on the current distribution of their genetic diversity. Habitat suitability under present and predicted future climate conditions was modeled for all species, to identify potential overlap zones between cultivated rice and its wild relatives, as well as to assess the expected impact of climate change on the *in situ* conservation status of the CWRs.

Both rainfed and irrigated rice cultivation is practiced in Colombia. Distribution data of both cultivation types were obtained from FEDEARROZ, the Colombian national rice federation (http://www.fedearroz.com.co). Suitability mapping was carried out separately for both rainfed and irrigated rice (236 and 349 presence cells, respectively), and posteriorly merged to obtain overall suitability maps of cultivated rice in Colombia. Presence data of the four CWRs collected during field sampling were complemented with records extracted from numerous sources (www.gbif.org; www.cwrdiversity.org; www.tropicos.org). Given the low number of presence points available for Colombia, we carried out model calibrations based on all species records available for Latin America, from Mexico to Argentina (Additional file 14: Figure S8). Record numbers per species are given in Additional file 15: Table S7.

We applied two different strategies for suitability modeling under past and future climate conditions. Model calibrations for projections to LGM climate conditions (only for CWR) were carried out at 2.5 arc minute resolution using only WorldClim climate layers (Hijmans et al. 2005) as explanatory variables. Model calibrations intended for projections to future climate scenarios (period 2040–2069; referred to as 2050s), were carried out at 30 arc sec resolution, using aside from climate layers also altitude, slope, aspect, terrain roughness, direction of water flow and soil type (FAO/IIASA/ISRIC/ISS-CAS/JRC 2012). Collinear explanatory variables were removed based on iterative calculations of variance inflation factors (VIF), retaining only variables with VIFs smaller than 5. The resulting sets of explanatory variables, as well as presence, background and absence points used for model calibrations are given in Additional file 15: Table S7.

Background points (an overall maximum of 10,000 and maximum one per grid cell) for LGM model calibrations were randomly selected from the area enclosed by a convex hull polygon constructed around all presence points and extended with a buffer corresponding to 10% of the polygon’s largest axis. For future model calibrations we additionally limited the selection of background points to areas of the extended convex hull intersected by the vegetation units from Olson et al. (Olson et al. 2001) with at least one species presence.

Modeling algorithms considered in the ensembles were maximum entropy (MAXENT), boosted regression trees (BRT, including a stepwise implementation), random forests (RF), generalized linear models (GLM; including stepwise selection of explanatory variables), generalized additive models (GAM; including stepwise selection of explanatory variables), multivariate adaptive regression splines (MARS), regression trees (RT), artificial neural networks (ANN), flexible discriminant analysis (FDA), support vector machines (SVM), and the BIOCLIM algorithm. As spatial autocorrelation among species presence points is known to bias model evaluations based on cross-validation, presence data permitting, we evaluated the ability of all individual modeling algorithms to cope with spatial autocorrelation by calculating calibrated Area Under Curve (cAUC) values and comparing these with a geographical null model (Hijmans 2012). We compared the cAUCs of each of the distribution models with the cAUCs of the geographical null model resulting from ten iterations, by means of Mann-Whitney tests. Only models that gave cAUC values that were significantly higher than the null model were retained in the ensemble model used for projections. Each ensemble combination was constructed as the weighted average of its individual composing models, using the cAUC values as weights. In cases where it was not possible to calculate cAUC values (i.e. when less than 10 presences available for model testing), we used AUC in a similar manner as described above, but included all models in the ensemble that yielded AUC values equal or higher than the null model.

To assess habitat suitability under LGM climate conditions we carried out projections to two climate models (MIROC and CCSM; (Braconnot et al. 2007)). For characterizing future climate conditions, we used 30 downscaled climate models for the period 2040–2069 based on the Representative concentration pathway (RCP) 4.5 scenario of greenhouse gas emissions, prepared for the Fifth Assessment IPCC report (CMIP5) and obtained from the WorldClim website (http://www.worldclim.org/CMIP5v1). The RCP4.5 scenario assumes that greenhouse gas emissions will peak around 2040 and will then decline. It is expected that under scenario global surface temperature change for the end of the 21st century is more likely than not to exceed 2 °C (Thomson et al. 2011).

We limited model projections to areas where suitability scores were at least as high as the score of the least suitable presence cell. This is considered a liberal approach in distribution modeling, as the likeliness of false positives and overestimation of suitable areas increases at lower thresholds. Since our purpose here was to identify areas where each of the species has a realistic chance of surviving and reproducing, either naturally (rice CWRs) or through human intervention (cultivated rice), potential overestimations are justified. In fact, the use of the highest possible threshold that includes all presence locations in the area identified as suitable might still be too conservative as there is no certainty that our presence data include representative samples at the extremes of each species’ fundamental niche. To obtain summarizing maps for the two LGM climate models we averaged the threshold-limited suitability maps constructed for both individual climate scenarios.

Future suitability maps (period 2040–2069) were limited to areas that were identified as suitable by at least half of all 30 possible threshold-limited climate projections. To identify areas where suitable habitat of cultivated rice (irrigated and rainfed) can realistically spatially overlap with that of its four wild relatives in Colombia, we overlaid maps of the combined current and future habitat suitability for cultivated rice and each of the wild relatives.

### Statistical analysis and genetic diversity mapping

For the allotetraploid species, diversity parameters H_E_ (expected heterozygosity) and G_ST_ (proportion of genetic diversity attributed to the differentiation among the populations) were estimated with ATetra v1.2 (Van Puyvelde et al. 2010). Due to the large number of partial heterozygotes present in the species analyzed (which significantly increases the number of iterative substitutions to generate the tetraploid genotypes), we carried out 10,000 Monte Carlo simulations for obtaining approximate diversity indices. The concept of partial heterozygote refers to the situation where for a given locus three alleles are observed and one of these (undetermined) is homozygous. For example, an ABC microsatellite pattern for one locus could represent a genotype of AABC, ABBC, or ABCC. For the diploid species, *O. glumaepatula*, the number of alleles per locus (A) and the estimators of H_E_, observed heterozygosity (H_O_), overall fixation index (F_IT_), fixation index or genetic differentiation of subpopulations (F_ST_), and inbreeding coefficient (F_IS_), were calculated with FSTAT v2.9.3 (Goudet 2001). Correlations between genetic and geographical distance matrices were assessed by means of Mantel tests with 10,000 permutations, using the R (R Development Core Team 2011) *ade4* package (Dray and Dufour 2007).

We carried out Principal Coordinates Analyses (PCoA) for visualizing genetic differentiation between and within species samples, using the R package *Polysat* (Clark and Jasieniuk 2011). Genetic distance matrices for both diploid and tetraploid species were based on the Bruvo distance (Bruvo et al. 2004) which is applicable to organisms of any ploidy level. The Bruvo distance is similar to band-sharing indices used with dominant data, but takes into account mutational distances between alleles, to address the fact that allele copy numbers are frequently unknown in polyploid microsatellite data (Clark and Jasieniuk 2011). PCoA of the diploid *O. glumaepatula* showed the same structure using either the Nei (not shown) or the Bruvo distance. We identified different genetic groups for each of the species by submitting the Bruvo distance matrices to hierarchical cluster analysis with bootstrap resampling (10,000 replicas) to provide indications of branch support, implemented in the R package *pvclust* (Suzuki and Shimodaira 2015). We tested different clustering methods and selected the method generating the highest cophenetic correlation coefficient, being the Unweighted Pair Group Method with Arithmetic Mean in all cases (cophenetic coefficients >0.92). Only clusters with branch support higher than 95% were considered. We additionally used STRUCTURE version 2.3.4 (Pritchard et al. 2000) to probabilistically assign individuals to genetic clusters (K) in each of the rice CWR, under admixture model assumptions and without consideration of sampling localities. For *O. alta*, *O. grandiglumis* and *O. glumaepatula* the number of groups (K) tested varied from 1 to 6, and for *O. latifolia* from 1 to 13. We used burnin periods of one million steps followed by ten million additional MCMC repetitions. For each value of K, we carried out 10 independent iterations. We used the method of Evanno et al. (2005) for detection of the most probable number of genetically homogeneous clusters (K), through use of the STRUCTURE HARVESTER software v0.6.94 (Dent and VonHoldt 2011) as implemented on the Web server http://taylor0.biology.ucla.edu/structureHarvester.

To visualize geographical patterns in SRR diversity of all species, we carried out grid-based spatial analyses at a resolution of 30 arc sec (~1 km at the equator). To obtain sufficient and more evenly distributed data points for constructing high resolution maps, we constructed circular neighborhoods of 5 arc minutes diameter (~10 km at the equator), following (Thomas et al. 2012). For each species we calculated values of allelic richness and Shannon diversity of individual raster cells based on all individuals included in the circular area of 5 arc minutes diameter. Calculation of parameters for tetraploid and diploid species was carried out with functions from the *Polysat* package and custom-made code for R (Thomas et al. 2012), respectively. The circular neighborhood diameter was chosen to allow comparison of the genetic profile of adjacent populations and hence detect spatial patterns at landscape level across the species distributions. All maps were edited in ArcMap v10 (ESRI).

## Additional files

Additional file 1: Table S1. Genetic diversity estimators for the tetraploid species *O. alta*, *O. grandiglumis* and *O. latifolia*. (DOCX 20 kb)

Additional file 2: Table S2. Genetic diversity estimators for the diploid species *O. glumaepatlula*. (DOCX 14 kb)

Additional file 3: Figure S1. Hierarchical cluster dendrograms based on the Bruvo and Nei (the latter for *O. glumaepatula* only) genetic distances and the Unweighted Pair Group Method with Arithmetic Mean. To types of p-values are provided to quantify branch support: the AU (Approximately Unbiased) p-value and BP (Bootstrap Probability) value. According to Suzuki and Shimodaira (2015) “*AU p-value, which is computed by multiscale bootstrap resampling, is a better approximation to unbiased p-value than BP value computed by normal bootstrap resampling*”. Clusters with branch support >95% are highlighted in boxes with different colors. (ZIP 572 kb)

Additional file 4: Figure S2. ΔK values of all rice wild relatives for different values of K tested, following Evanno et al. (2005), implemented in the STRUCTURE HARVESTER software (Dent and VonHoldt 2011). (TIF 1302 kb)

Additional file 5: Figure S3. Habitat suitability of *O. alta* during the Last Glacial Maximum (~21,000BP), compared with the distribution of genetic clusters and Shannon diversity index of sampled populations. (TIF 1687 kb)

Additional file 6: Figure S4. Habitat suitability of *O. glumaepatula* during the Last Glacial Maximum (~21,000BP), compared with the distribution of genetic clusters and Shannon diversity index of sampled populations. (TIF 1751 kb)

Additional file 7: Figure S5. Habitat suitability of *O. grandiglumis* during the Last Glacial Maximum (~21,000BP), compared with the distribution of genetic clusters and Shannon diversity index of sampled populations. (TIF 1731 kb)

Additional file 8: Table S3. Results of Mantel tests for all rice CWR between genetic and geographical distances for all individuals sampled and individuals per genetic cluster. (DOCX 14 kb)

Additional file 9: Figure S6. Predicted changes in habitat suitability from present to the 2050s of cultivated rice *Oryza sativa*, considering both rainfed and irrigated rice. (TIF 4570 kb)

Additional file 10: Table S4. Metrics obtained from genetic diversity studies of *O. glumaepatula* in Latin America. (DOCX 12 kb)

Additional file 11: Figure S7. Details of panicles (top), ligules and auricles (bottom) of (A) *Oryza latifolia* with flower size smaller than 7 mm; (B) *Oryza grandiglumis* with approximately equal-sized sterile and fertile lemmas; and (C) *Oryza alta* with flower size larger than 7 mm (photo credits: Oscar Andrés Estrada). (DOCX 180 kb)

Additional file 12: Table S5. List of analyzed *Oryza* spp. samples with genetic characterization data. (CSV 74 kb)

Additional file 13: Table S6. PCR primer sequences, allele size ranges and chromosome position on the Nipponbare genome of the SSR loci used in this study. (XLSX 12 kb)

Additional file 14: Figure S8. Distribution of species records used in suitability model calibrations. (TIF 1695 kb)

Additional file 15: Table S7. Suitability model statistics and metrics. (DOCX 17 kb)

## References

[CR1] Aggarwal RK, Brar DS, Huang N, Khush GS (1996). Differentiation within CCDD genome species in the genus Oryza as revealed by total genomic hybridization and RFLP analysis. Rice Genet Newsl.

[CR2] Aggarwal RK, Brar DS, Khush GS (1997). Two new genomes in the Oryza complex identified on the basis of molecular divergence analysis using total genomic DNA hybridization. Mol Gen Genet.

[CR3] Aggarwal RK, Brar DS, Nandi S (1999). Phylogenetic relationships among Oryza species revealed by AFLP markers. Theor Appl Genet.

[CR4] Akimoto M, Shimamoto Y, Morishima H (1998). Population genetic structure of wild Oryza glumaepatula distributed in the Amazon flood area influenced by its life history traits. Mol Ecol.

[CR5] Arrieta-Espinoza G, Sánchez E, Vargas S (2005). The weedy rice complex in Costa Rica. I. Morphological Study of relationships between commercial rice varieties, wild Oryza relatives and weedy types. Genet Resour Crop Evol.

[CR6] Atwell BJ, Wang H, Scafaro AP (2014). Could abiotic stress tolerance in wild relatives of rice be used to improve Oryza sativa?. Plant Sci.

[CR7] Bao Y, Ge S (2003). Identification of Oryza species with the CD genome based on the RFLP analysis of nuclear ribosomal ITS sequences. Acta Bot Sin.

[CR8] Bassam B, Caetano-Anollés G, Gresshoff P (1991). Fast and sensitive silver staining of DNA in polyacrylamide gels. Anal Biochem.

[CR9] Braconnot P, Otto-bliesner B, Harrison S (2007). Results of PMIP2 coupled simulations of the mid-holocene and last glacial maximum – part 2: feedbacks with emphasis on the location of the ITCZ and mid- and high latitudes heat budget. Clim Past.

[CR10] Brar D, Singh K, Kole C (2011). Oryza. Wild crop relatives: Genomic and breeding resources: cereals.

[CR11] Brondani RPV, Zucchi MI, Brondani C (2005). Genetic structure of wild rice Oryza glumaepatula populations in three Brazilian biomes using microsatellite markers. Genetica.

[CR12] Bruvo R, Michiels NK, D’souza TG, Schulen-Burg H (2004). A simple method for the calculation of microsatellite genotype distances irrespective of ploidy level. Mol Ecol.

[CR13] Buso GSC, Rangel PH, Ferreira ME (1998). Analysis of genetic variability of South American wild rice populations (Oryza glumaepatula) with isozymes and RAPD markers. Mol Ecol.

[CR14] Castañeda-Álvarez NP, Khoury CK, Achicanoy HA, et al. (2016) Global conservation priorities for crop wild relatives. Nat Plants 16022. doi: 10.1038/nplants.2016.22 10.1038/nplants.2016.22 27249561

[CR15] CERA (Center for Environmental Risk Assessment) (2012) GM Crop Database. ILSI Research Foundation, Washington D.C. http://cera-gmc.org/GMCropDatabase. Accessed 20 June 2015

[CR16] Chen LJ, Lee DS, Song ZP (2004). Gene flow from cultivated rice (Oryza sativa) to its weedy and wild relatives. Ann Bot.

[CR17] Chen J, Kallman T, Ma X (2012). Disentangling the roles of history and local selection in shaping clinal variation of allele frequencies and gene expression in Norway spruce (Picea abies). Genetics.

[CR18] Clark LV, Jasieniuk M (2011). Polysat: an R package for polyploid microsatellite analysis. Mol Ecol Resour.

[CR19] Dempewolf H, Eastwood RJ, Guarino L (2014). Adapting agriculture to climate change: a global initiative to collect, conserve, and use crop wild relatives. Agroecol Sustain Food Syst.

[CR20] Dent EA, VonHoldt BM (2011). STRUCTURE HARVESTER: a website and program for visualizing STRUCTURE output and implementing the Evanno method. Conserv Genet Resour.

[CR21] Dray S, Dufour AB (2007). The ade4 package: implementing the duality diagram for ecologists. J Stat Softw.

[CR22] Ellstrand NC (2003). Dangerous liaisons? When cultivated plants mate with their wild relatives.

[CR23] Evanno G, Regnaut S, Goudet J (2005). Detecting the number of clusters of individuals using the software STRUCTURE: a simulation study. Mol Ecol.

[CR24] FAO/IIASA/ISRIC/ISS-CAS/JRC (2012) Harmonized world soil database (version 1.2). FAO/IIASA, Rome, Luxemburg

[CR25] Federici MT, Shcherban AB, Capdevielle F, et al (2002) Analysis of genetic diversity in the Oryza officinalis complex. Electron J Biotechnol 5:0–0. doi: 10.2225/vol5-issue2-fulltext-9

[CR26] Fuller DQ, Sato YI, Castillo C (2010). Consilience of genetics and archaeobotany in the entangled history of rice. Archaeol Anthropol Sci.

[CR27] Galluzzi G, Dufour D, Thomas E (2015). An Integrated Hypothesis on the Domestication of Bactris gasipaes. PLoS One.

[CR28] Gao L, Schaal BA, Zhang C (2002). Assessment of population genetic structure in common wild rice Oryza rufipogon Griff. using microsatellite and allozyme markers. Theor Appl Genet.

[CR29] Garcia MA, Altieri MA (2005). Transgenic Crops: Implications for Biodiversity and Sustainable Agriculture. Bull Sci Technol Soc.

[CR30] Ge S, Sang T, Lu B-R, Hong D-Y (1999). Phylogeny of rice genomes with emphasis on origins of allotetraploid species. Proc Natl Acad Sci.

[CR31] Goudet J (2001) FSTAT, a program to estimate and test gene diversities and fixation indices (version 2.9.3). Available at: https://www.unil.ch/dee/en/home/menuinst/softwares--dataset/softwares/fstat.html

[CR32] Hajjar R, Hodgkin T (2007). The use of wild relatives in crop improvement: a survey of developments over the last 20 years. Euphytica.

[CR33] Hedrick PW (2005). A standardized genetic differentiation measure. Evolution.

[CR34] Hijmans RJ (2012). Cross-validation of species distribution models: removing spatial sorting bias and calibration with a null model. Ecology.

[CR35] Hijmans RJ, Cameron SE, Parra JL (2005). Very high resolution interpolated climate surfaces for global land areas. Int J Climatol.

[CR36] Huang P, Schaal B a (2012). Association between the geographic distribution during the last glacial maximum of Asian wild rice, Oryza rufipogon (Poaceae), and its current genetic variation. Am J Bot.

[CR37] Jena KK (2010). The species of the genus Oryza and transfer of useful genes from wild species into cultivated rice, O. sativa. Breed Sci.

[CR38] Joshi SP, Gupta VS, Aggarwal RK (2000). Genetic diversity and phylogenetic relationship as revealed by inter simple sequence repeat (ISSR) polymorphism in the genus Oryza. Theor Appl Genet.

[CR39] Jost L (2008). GST and its relatives do not measure differentiation. Mol Ecol.

[CR40] Kanya JI, Kinyamario JI, Amugune NO, Hauser TP (2009). Dispersal distance of rice (Oryza Sativa L.) pollen at the Tana River delta in the coast province, Kenya. African Journal Biotechnol.

[CR41] Karasawa MMG, Vencovsky R, Silva CM (2007). Genetic structure of Brazilian wild rice (Oryza glumaepatula Steud., Poaceae) populations analyzed using microsatellite markers. Genet Mol Biol.

[CR42] Karasawa MMG, Vencovsky R, Silva CM (2007). Mating system of Brazilian Oryza glumaepatula populations studied with microsatellite markers. Ann Bot.

[CR43] Karasawa MMG, Vencovsky R, Silva CM (2012). Comparison of microsatellites and isozymes in genetic diversity studies of Oryza glumaepatula (Poaceae) populations. Rev Biol Trop.

[CR44] Khoury CK, Castañeda-Alvarez NP, Achicanoy H a (2015). Crop wild relatives of pigeonpea [Cajanus cajan (L.) Millsp.]: distributions, ex situ conservation status, and potential genetic resources for abiotic stress tolerance. Biol Conserv.

[CR45] Kindt R, Coe R (2005). A manual and software for common statistical methods for ecological biodiversity studies.

[CR46] Knowles LL, Richards CL (2005). Importance of genetic drift during Pleistocene divergence as revealed by analyses of genomic variation. Mol Ecol.

[CR47] McCouch S, Teytelman L, Xu Y (2002). Development and mapping of 2240 new SSR markers for rice (Oryza sativa L.). DNA Res.

[CR48] Meirmans PG, Hedrick PW (2011). Assessing population structure: FST and related measures. Mol Ecol Resour.

[CR49] Messeguer J, Fogher C, Guiderdoni E (2001). Field assessment of gene flow from transgenic to cultivated rices (Oryza sativa L.) using a herbicide resistance genes as tracer marker. Theor Appl Genet.

[CR50] Mittell EA, Nakagawa S, Hadfield JD (2015) Are molecular markers useful predictors of adaptive potential? Ecol Lett 18:n/a-n/a. doi: 10.1111/ele.12454 10.1111/ele.12454 25989024

[CR51] Morjan CL, Rieseberg LH (2004). How species evolve collectively: implications of gene flow and selection for the spread of advantageous alleles. Mol Ecol.

[CR52] Naredo M, Juliano A, Lu B, Jackson M (1997). Hybridization of AA genome rice species from Asia and Australia, I: crosses and development of hybrids. Genet Resour Crop Evol.

[CR53] Naredo M, Juliano A, Lu B, Jackson M (1998). Taxonomic status of Oryza glumaepatula Steud, II: hybridization between new world diploids and AA genome species from Asia and Australia. Genet Resour Crop Evol.

[CR54] Nei M (1973). Analysis of gene diversity in subdivided populations. Proc Natl Acad Sci.

[CR55] Niroula RK, Pucciariello C, Ho VT (2012). SUB1A-dependent and -independent mechanisms are involved in the flooding tolerance of wild rice species. Plant J.

[CR56] Oka HI (1988). Origin of cultivated rice.

[CR57] Olson DM, Dinerstein E, Wikramanaya ED (2001). Terrestrial ecoregions of the world: a new map of life on earth. Bioscience.

[CR58] Pak JH, Oh JS, Kim HJ (2013). Development of near-isogenic transgenic rice lines harboring wild rice (Oryza grandiglumis)-derived fungal resistance gene (OgPR1). Plant Breed Biotechnol.

[CR59] Piálek J, Barton NH (1997). The spread of an advantageous allele across a barrier: the effects of random drift and selection against heterozygotes. Genetics.

[CR60] Pritchard JK, Stephens M, Donnelly P (2000). Inference of population structure using multilocus genotype data. Genetics.

[CR61] Pu D, Shi M, Wu Q (2014). Flower-visiting insects and their potential impact on transgene flow in rice. J Appl Ecol.

[CR62] Quesada T, Lobo J, Espinoza AM (2002). Isozyme diversity and analysis of the mating system of the wild rice Oryza latifolia Desv. in Costa Rica. Genet Resour Crop Evol.

[CR63] R Development Core Team (2011). R: A language and environment for statistical computing. version 2.14.

[CR64] Rong J, Xia H, Zhu Y (2004). Asymmetric gene flow between traditional and hybrid rice varieties (Oryza sativa) indicated by nuclear simple sequence repeats and implications for germplasm conservation. New Phytol.

[CR65] Rong J, Song Z, Su J (2005). Low frequency of transgene flow from Bt/CpTI rice to its non-transgenic counterparts planted at close spacing. New Phytol.

[CR66] Rong J, Lu BR, Song Z (2007). Dramatic reduction of crop-to-crop gene flow within a short distance from transgenic rice fields. New Phytol.

[CR67] Sanchez PL, Wing RA, Brar DS, Zhang Q, Wing RA (2013). The wild relative of rice: genomes and genomics. Genetics and genomics of rice, plant gene.

[CR68] Schaal BA, Hayworth DA, Olsen KM (1998). Phylogeographic studies in plants: problems and prospects. Mol Ecol.

[CR69] Snow A, Andow D, Gepts P (2005). Genetically engineered organisms and the environment: Current status and recommendations. Ecol Appl.

[CR70] Song Z, Lu B, Chen J (2001). A study of pollen viability and longevity in Oryza rufipogon, O. sativa and their hybrid. Int Rice Res Notes.

[CR71] Song Z, Lu B, Zhu Y, Chen J (2002). Pollen competition between cultivated and wild rice species (Oryza sativa and O. rufipogon). New Phytol.

[CR72] Song ZP, Lu B-R, Zhu YG, Chen JK (2003). Gene flow from cultivated rice to the wild species Oryza rufipogon under experimental field conditions. New Phytol.

[CR73] Stewart CN, Halfhill MD, Warwick SI (2003). Transgene introgression from genetically modified crops to their wild relatives. Nat Rev Genet.

[CR74] Sun GL, Diaz O, Salomon B, Von Bothmer R (1998). Microsatellite variation and its comparison with allozyme and RAPD variation in Elymus fibrosus (Schrenk) Tzvel. (Poaceae). Hereditas.

[CR75] Suzuki R, Shimodaira H (2015) pvclust: hierarchical clustering with p-values via multiscale bootstrap resampling. R package version 2.0-0. http://CRAN.R-project.org/package=pvclust

[CR76] Thomas E, van Zonneveld M, Loo J (2012). Present spatial diversity patterns of theobroma cacao L. in the neotropics reflect genetic differentiation in pleistocene refugia followed by human-influenced dispersal. PLoS One.

[CR77] Thomas E, Alcazar Caicedo C, McMichael CH (2015). Uncovering spatial patterns in the natural and human history of Brazil nut (Bertholletia excelsa) across the Amazon basin. J Biogeogr.

[CR78] Thomas E, Ramirez M, Van Zonneveld M, Maxted N, Dulloo E, Ford-LLoyd BV (2016). An assessment of the conservation status of Mesoamerican crop species and their wild relatives in light of climate change. Enhancing crop genepool utilization: capturing wild relative and landrace diversity for crop improvement.

[CR79] Thomas E, Gil Tobón C, Gutierrez JP, Alcazar Caicedo C, Moscoso Higuita LG, Becerra LA, Loo J, Gonzales MA (2017) Genetic diversity of Enterolobium cyclocarpum in Colombian seasonally dry tropical forest: implications for conservation and restoration. Biodivers Conserv 26:825–842

[CR80] Thomson AM, Calvin KV, Smith SJ (2011). RCP4.5: a pathway for stabilization of radiative forcing by 2100. Clim Change.

[CR81] Van Puyvelde K, Van Geert A, Triest L (2010). ATETRA, a new software program to analyse tetraploid microsatellite data: comparison with TETRA and TETRASAT. Mol Ecol Resour.

[CR82] Vaughan DA (1989) The genus Oryza L. Current status of taxonomy. IRRI Research Paper Series No. 138, Manila

[CR83] Vaughan DA, Nanda JS, Sharma SD (2003). Revised key to species in genus Oryza - appendix 1. Monograph on genus Oryza.

[CR84] Vaughan D, Morishima H, Kadowaki K (2003). Diversity in the Oryza genus. Curr Opin Plant Biol.

[CR85] Veasey EA, Cardin DC, Silva RM (2008). Assessing the genetic structure of Oryza glumaepatula populations with isozyme markers. Brazilian Arch Biol Technol.

[CR86] Veasey EA, Da Silva EF, Schammass EA (2008). Morphoagronomic genetic diversity in American wild rice species. Brazilian Arch Biol Technol.

[CR87] Veasey EA, Bressan EDA, Zucchi MI (2011). Genetic diversity of American wild rice species. Sci Agric.

[CR88] Villafañe C, Estrada OA, Lentini Z, Hodson de Jaramillo E, Carrizosa P (2007). Diagnóstico y fortalecimiento de la línea base del conocimiento del género Oryza (arroz) en Colombia, un aporte para la toma de decisiones en el ámbito de bioseguridad. Desarrollo de capacidades para evaluación y gestión de riesgos y monitoreo de organismos genéticamente modificados (OGM). Tomo I. Resultados de proyectos específicos.

[CR89] Vincent H, Wiersema J, Kell S (2013). A prioritized crop wild relative inventory to help underpin global food security. Biol Conserv.

[CR90] Waltari E, Hijmans RJ, Peterson T (2007). Locating pleistocene refugia: comparing phylogeographic and ecological niche model predictions. PLoS One.

[CR91] Wang ZY, Second G, Tanksley SD (1992). Polymorphism and phylogenetic relationships among species in the genus Oryza as determined by analysis of nuclear RFLPs. Theor Appl Genet.

[CR92] Wang F, Yuan Q-H, Shi L (2006). A large-scale field study of transgene flow from cultivated rice (Oryza sativa) to common wild rice (O. rufipogon) and barnyard grass (Echinochloa crusgalli). Plant Biotechnol J.

[CR93] Yao K, Hu N, Chen W (2008). Establishment of a rice transgene flow model for predicting maximum distances of gene flow in southern China. New Phytol.

[CR94] Zamora A, Barboza C, Lobo J, Espinoza AM (2003). Diversity of native rice (Oryza Poaceae:) species of Costa Rica. Genet Resour Crop Evol.

