# Supplementary material for: Distribution, genetic diversity and potential spatiotemporal scale of alien gene flow in crop wild relatives of rice (Oryza spp.) in Colombia
Source: Rice (N Y). 2017 Apr 18;10:13. doi: 10.1186/s12284-017-0150-9 (PMC5395511; doi:10.1186/s12284-017-0150-9)
Supplement: Supplementary file 1 — Genetic diversity estimators for the tetraploid species O. alta, O. grandiglumis and O. latifolia. (DOCX 20 kb) [file 12284_2017_150_MOESM1_ESM.docx]

| **Additional file 6: Table S4**. Genetic diversity estimators for the tetraploid species *O. alta, O. grandiglumis* and *O. latifolia*. | | | | | | | |
| --- | --- | --- | --- | --- | --- | --- | --- |
| **Species** | **Locus** | **A** | **H_E_** | | | | **H_E_** |
|  |  |  | **P1** | **P2** | **P3** | **P4** |  |
| ***O. alta***  **(n = 49)** | **RM 224** | 5 | 0.185 | 0.735 |  |  | 0.460 |
|  | **RM60** | 10 | 0.627 | 0.724 |  |  | 0.675 |
|  | **RM 234** | 4 | 0.504 | 0.681 |  |  | 0.593 |
|  | **RM 451** | 2 | 0.000 | 0.000 |  |  | 0.000 |
|  | **RM 332** | 1 | 0.000 | 0.000 |  |  | 0.000 |
|  | **RM 408** | 4 | 0.612 | 0.652 |  |  | 0.632 |
|  | **RM 484** | 2 | 0.096 | 0.000 |  |  | 0.048 |
|  | **RM 169** | 8 | 0.504 | 0.747 |  |  | 0.625 |
|  | **RM479** | 5 | 0.645 | 0.709 |  |  | 0.677 |
|  | **RM536** | 3 | 0.550 | 0.501 |  |  | 0.526 |
|  | **RM254** | 2 | 0.000 | 0.384 |  |  | 0.192 |
|  | **Mean** | **4.2** | **0.339** | **0.467** |  |  | **0.403** |
|  |  |  |  |  |  |  |  |
| ***O. grandiglumis***  **(n = 37)** | **RM 224** | 6 | 0.503 | 0.690 |  |  | 0.596 |
|  | **RM60** | 2 | 0.000 | 0.502 |  |  | 0.251 |
|  | **RM 234** | 5 | 0.638 | 0.502 |  |  | 0.570 |
|  | **RM 451** | 3 | 0.000 | 0.248 |  |  | 0.124 |
|  | **RM 332** | 5 | 0.558 | 0.502 |  |  | 0.530 |
|  | **RM 408** | 4 | 0.408 | 0.673 |  |  | 0.540 |
|  | **RM 484** | 3 | 0.000 | 0.671 |  |  | 0.336 |
|  | **RM 169** | 6 | 0.630 | 0.782 |  |  | 0.706 |
|  | **RM479** | 5 | 0.580 | 0.502 |  |  | 0.541 |
|  | **RM536** | 2 | 0.000 | 0.502 |  |  | 0.251 |
|  | **RM254** | 2 | 0.500 | 0.000 |  |  | 0.250 |
|  | **Mean** | **3.9** | **0.347** | **0.507** |  |  | **0.427** |
|  |  |  |  |  |  |  |  |
| ***O. latifolia***  **(n = 175)** | **RM 224** | 20 | 0.564 | 0.672 | 0.866 | 0.844 | 0.737 |
|  | **RM60** | 13 | 0.657 | 0.502 | 0.703 | 0.705 | 0.642 |
|  | **RM 234** | 14 | 0.675 | 0.558 | 0.678 | 0.712 | 0.656 |
|  | **RM 451** | 4 | 0.468 | 0.000 | 0.491 | 0.501 | 0.365 |
|  | **RM 332** | 5 | 0.515 | 0.502 | 0.171 | 0.577 | 0.441 |
|  | **RM 408** | 10 | 0.834 | 0.502 | 0.826 | 0.657 | 0.705 |
|  | **RM 484** | 9 | 0.764 | 0.000 | 0.556 | 0.760 | 0.520 |
|  | **RM 169** | 21 | 0.836 | 0.502 | 0.732 | 0.817 | 0.722 |
|  | **RM479** | 13 | 0.809 | 0.502 | 0.765 | 0.656 | 0.683 |
|  | **RM536** | 3 | 0.499 | 0.502 | 0.500 | 0.626 | 0.532 |
|  | **RM254** | 5 | 0.000 | 0.000 | 0.415 | 0.558 | 0.243 |
|  | **Mean** | **10.6** | **0.602** | **0.386** | **0.609** | **0.674** | **0.568** |
| A = number of alleles per locus, H_E_ = expected heterozygosity | | | | | | | |
