# Supplementary material for: Distribution, genetic diversity and potential spatiotemporal scale of alien gene flow in crop wild relatives of rice (Oryza spp.) in Colombia
Source: Rice (N Y). 2017 Apr 18;10:13. doi: 10.1186/s12284-017-0150-9 (PMC5395511; doi:10.1186/s12284-017-0150-9)
Supplement: Supplementary file 2 — Genetic diversity estimators for the diploid species O. glumaepatlula. (DOCX 14 kb) [file 12284_2017_150_MOESM2_ESM.docx]

| **Additional file 7: Table S5**. Genetic diversity estimators for the diploid species *O. glumaepatlula*. | | | | | | | | | |
| --- | --- | --- | --- | --- | --- | --- | --- | --- | --- |
| **Species** | **Locus** | **A** | **H_E_** | | **H_O_** | **H_E_** | **F_IT_** | **F_ST_** | **F_IS_** |
|  |  |  | **P1** | **P2** |  |  |  |  |  |
| ***O. glumaepatula***  **(n = 25)** | **RM224** | 4 | 0.000 | 0.548 | 0.500 | 0.281 | 0.235 | 0.579 | -0.819 |
|  | **RM060** | 1 | 0.000 | 0.000 | 0.000 | 0.000 | 0.000 | 0.000 | 0.000 |
|  | **RM234** | 4 | 0.000 | 0.607 | 0.048 | 0.391 | 0.924 | 0.495 | 0.849 |
|  | **RM451** | 3 | 0.000 | 0.524 | 0.000 | 0.343 | 1.000 | 0.552 | 1.000 |
|  | **RM332** | 5 | 0.500 | 0.545 | 0.524 | 0.604 | 0.858 | 0.386 | 0.768 |
|  | **RM408** | 2 | 0.000 | 0.476 | 0.381 | 0.253 | -0.245 | 0.215 | -0.586 |
|  | **RM484** | 2 | 0.000 | 0.000 | 0.000 | 0.000 | 1.000 | 1.000 | 1.000 |
|  | **RM169** | 2 | 0.000 | 0.000 | 0.000 | 0.000 | 1.000 | 1.000 | 1.000 |
|  | **RM479** | 2 | 0.000 | 0.379 | 0.048 | 0.241 | 0.736 | -0.090 | 0.758 |
|  | **RM536** | 2 | 0.000 | 0.095 | 0.000 | 0.062 | 1.000 | 0.907 | 1.000 |
|  | **RM254** | 3 | 0.000 | 0.179 | 0.048 | 0.110 | 0.917 | 0.839 | 0.483 |
|  | **Mean** | **2.7** | **0.045** | **0.304** | **0.141** | **0.208** | **0.784** | **0.655** | **0.374** |

A = number of alleles per locus, H_E_ = expected heterozygosity, H_O_ = observed heterozygosity, F_IS_= inbreeding coefficient; F_IT_ = overall inbreeding coefficient of an individual relative to the total population, F_ST=_ proportion of the total genetic variance contained in a subpopulation (the S subscript) relative to the total genetic variance
