# Supplementary figures and images for: Distribution, genetic diversity and potential spatiotemporal scale of alien gene flow in crop wild relatives of rice (Oryza spp.) in Colombia
Source: Rice (N Y). 2017 Apr 18;10:13. doi: 10.1186/s12284-017-0150-9 (PMC5395511; doi:10.1186/s12284-017-0150-9)

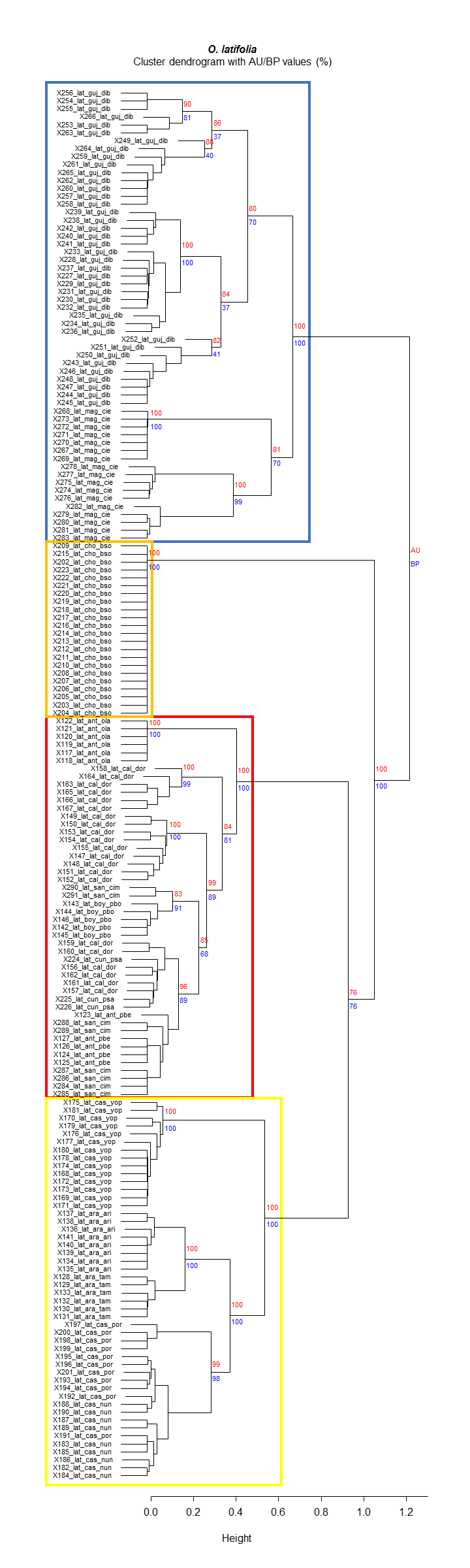

Supplement: Supplementary file 3 — Hierarchical cluster dendrograms based on the Bruvo and Nei (the latter for O. glumaepatula only) genetic distances and the Unweighted Pair Group Method with Arithmetic Mean. To types of p-values are provided to quantify branch support: the AU (Approximately Unbiased) p-value and BP (Bootstrap Probability) value. According to Suzuki and Shimodaira (2015) “AU p-value, which is computed by multiscale bootstrap resampling, is a better approximation to unbiased p-value than BP value computed by normal bootstrap resampling”. Clusters with branch support >95% are highlighted in boxes with different colors. (ZIP 572 kb) [file 12284_2017_150_MOESM3_ESM.zip › Fig S3.TIF]

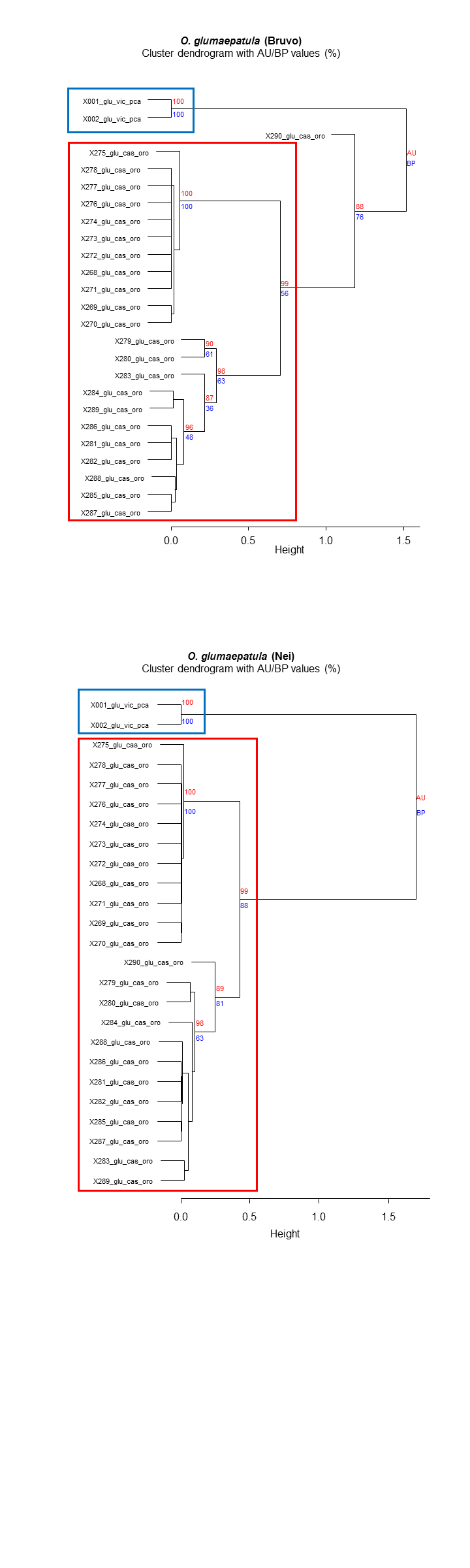

Supplement: Supplementary file 3 — Hierarchical cluster dendrograms based on the Bruvo and Nei (the latter for O. glumaepatula only) genetic distances and the Unweighted Pair Group Method with Arithmetic Mean. To types of p-values are provided to quantify branch support: the AU (Approximately Unbiased) p-value and BP (Bootstrap Probability) value. According to Suzuki and Shimodaira (2015) “AU p-value, which is computed by multiscale bootstrap resampling, is a better approximation to unbiased p-value than BP value computed by normal bootstrap resampling”. Clusters with branch support >95% are highlighted in boxes with different colors. (ZIP 572 kb) [file 12284_2017_150_MOESM3_ESM.zip › Fig S3 continued2.TIF]

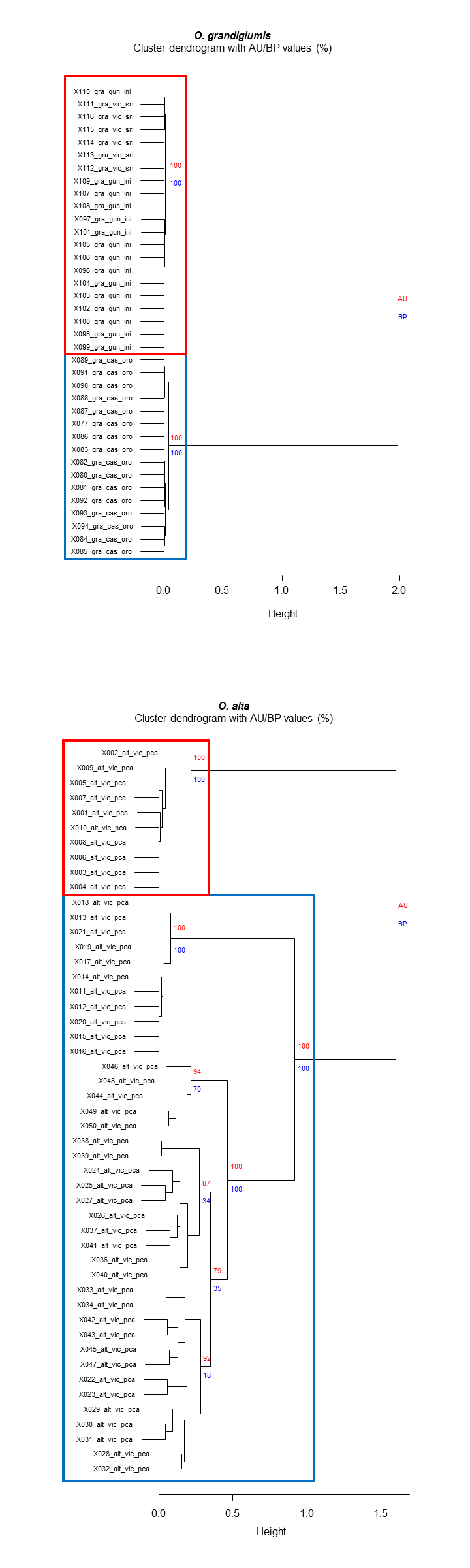

Supplement: Supplementary file 3 — Hierarchical cluster dendrograms based on the Bruvo and Nei (the latter for O. glumaepatula only) genetic distances and the Unweighted Pair Group Method with Arithmetic Mean. To types of p-values are provided to quantify branch support: the AU (Approximately Unbiased) p-value and BP (Bootstrap Probability) value. According to Suzuki and Shimodaira (2015) “AU p-value, which is computed by multiscale bootstrap resampling, is a better approximation to unbiased p-value than BP value computed by normal bootstrap resampling”. Clusters with branch support >95% are highlighted in boxes with different colors. (ZIP 572 kb) [file 12284_2017_150_MOESM3_ESM.zip › Fig S3 continued1.TIF]

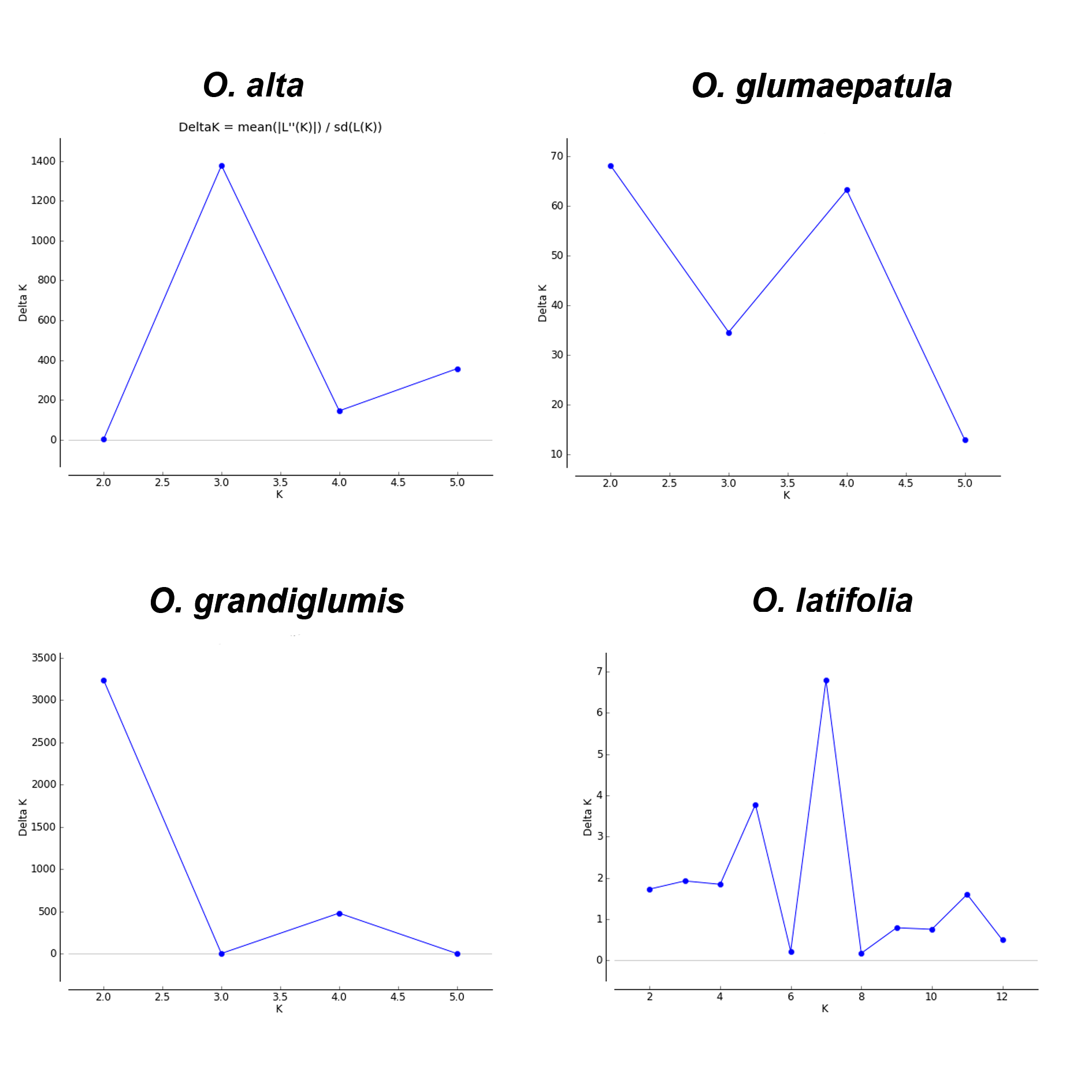

Supplement: Supplementary file 4 — ΔK values of all rice wild relatives for different values of K tested, following Evanno et al. (2005), implemented in the STRUCTURE HARVESTER software (Dent and VonHoldt 2011). (TIF 1302 kb) [file 12284_2017_150_MOESM4_ESM.tif]

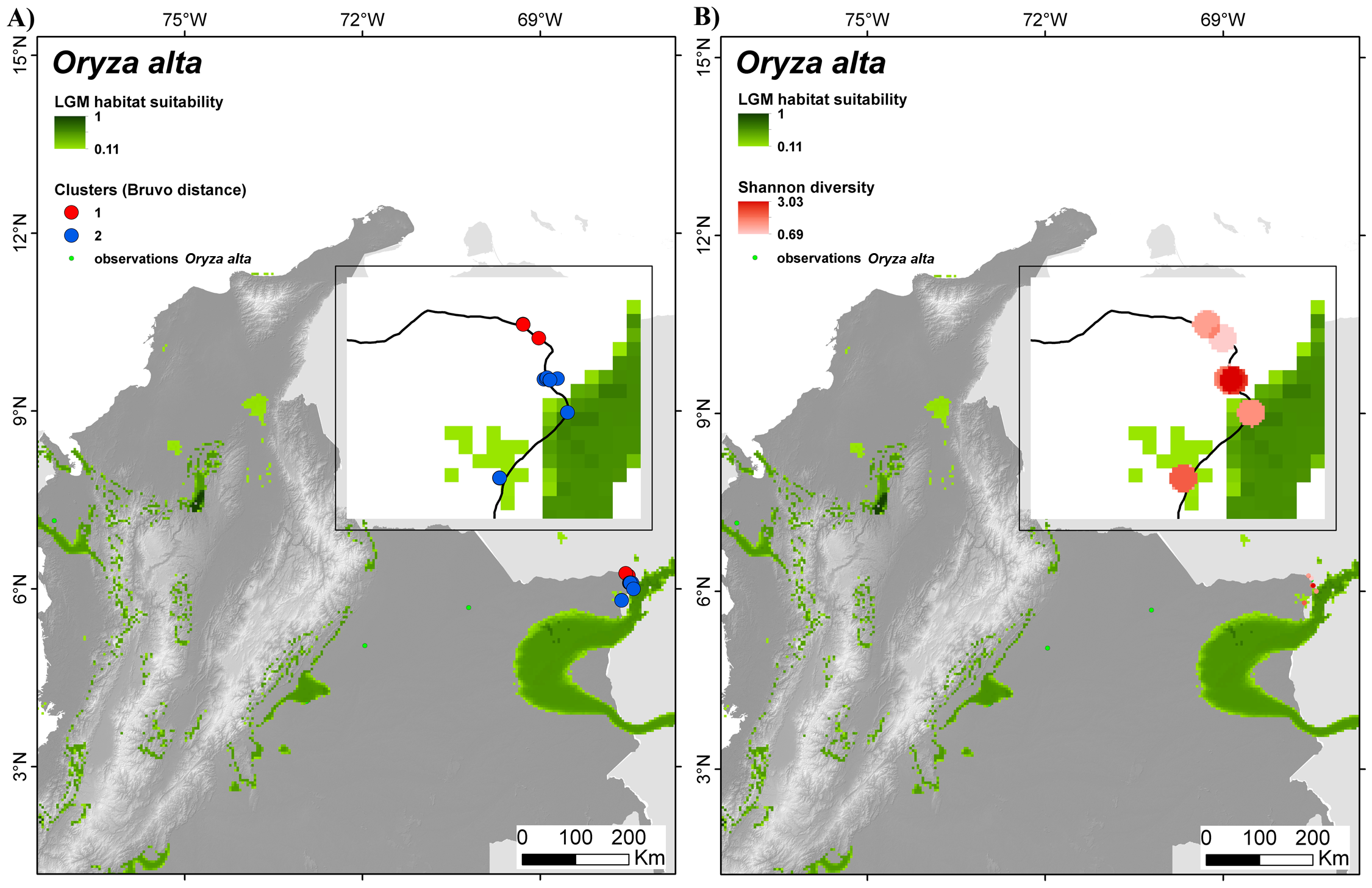

Supplement: Supplementary file 5 — Habitat suitability of O. alta during the Last Glacial Maximum (~21,000BP), compared with the distribution of genetic clusters and Shannon diversity index of sampled populations. (TIF 1687 kb) [file 12284_2017_150_MOESM5_ESM.tif]

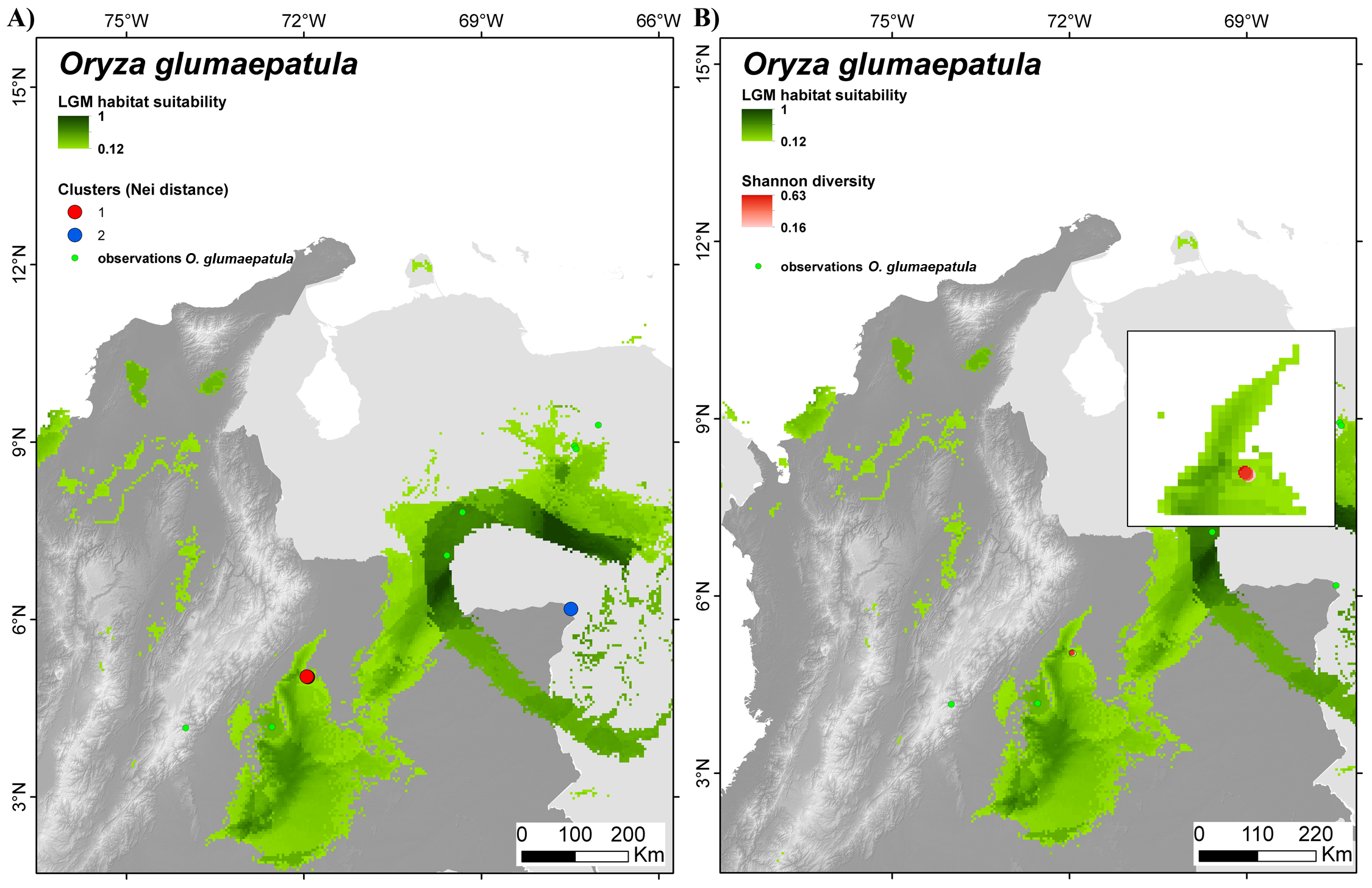

Supplement: Supplementary file 6 — Habitat suitability of O. glumaepatula during the Last Glacial Maximum (~21,000BP), compared with the distribution of genetic clusters and Shannon diversity index of sampled populations. (TIF 1751 kb) [file 12284_2017_150_MOESM6_ESM.tif]

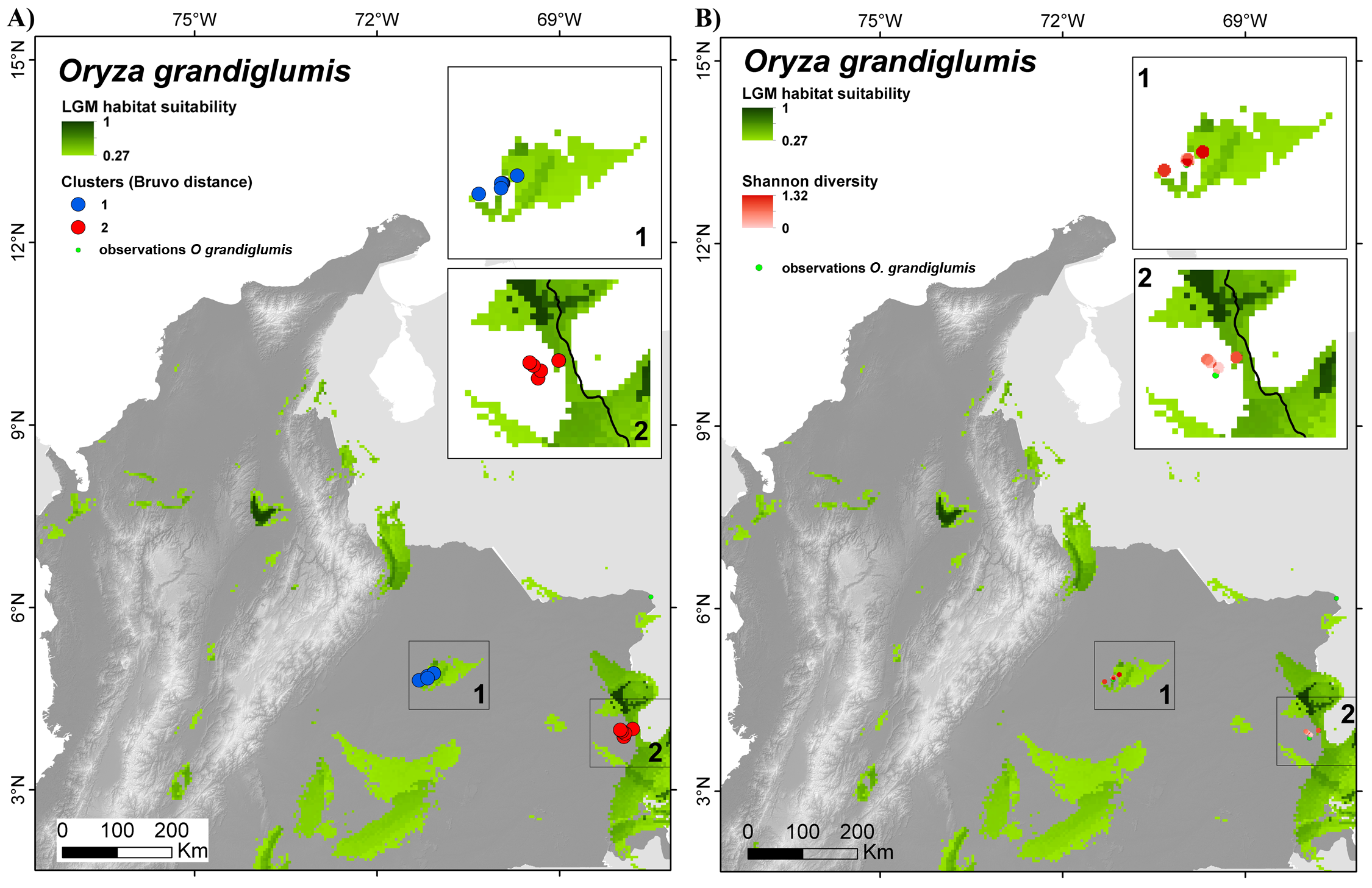

Supplement: Supplementary file 7 — Habitat suitability of O. grandiglumis during the Last Glacial Maximum (~21,000BP), compared with the distribution of genetic clusters and Shannon diversity index of sampled populations. (TIF 1731 kb) [file 12284_2017_150_MOESM7_ESM.tif]

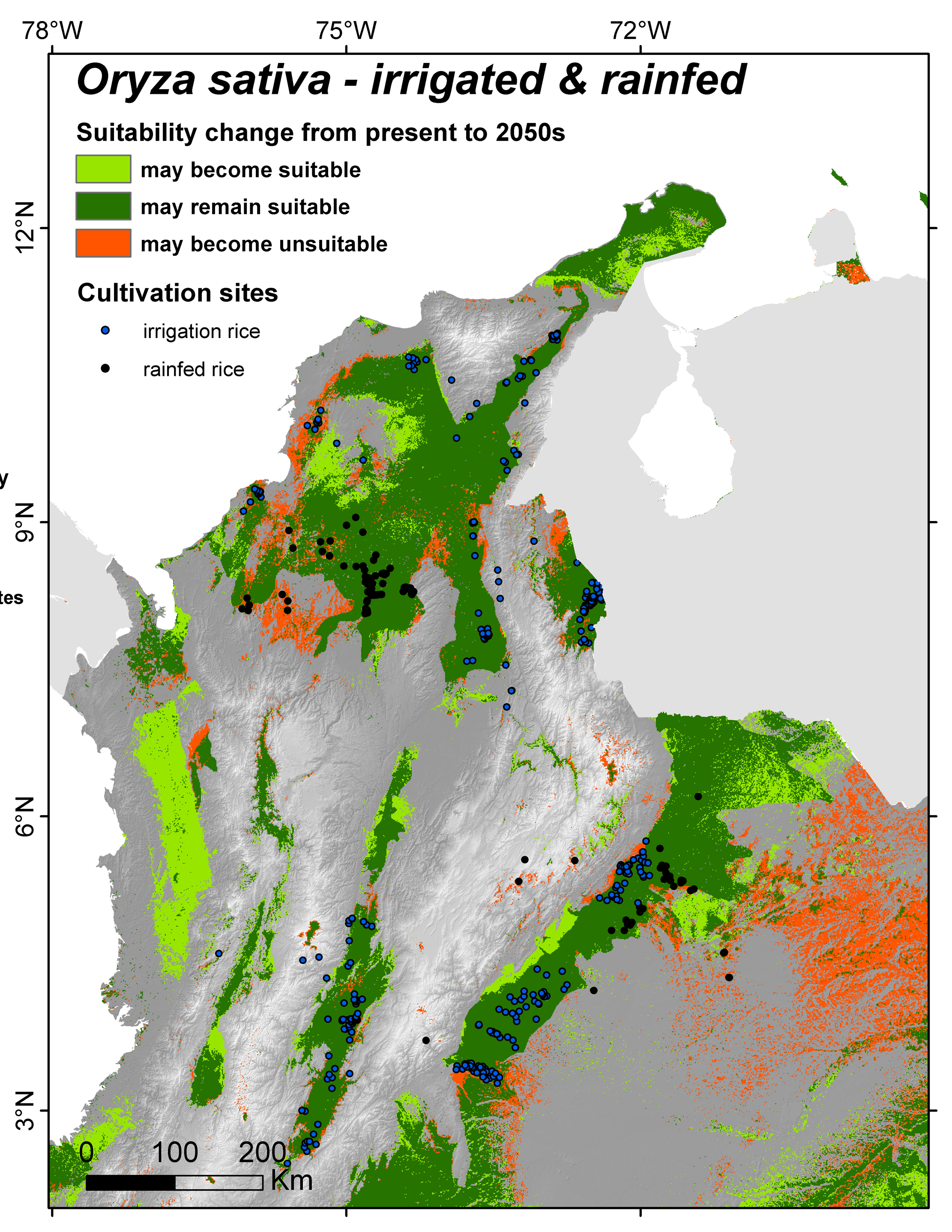

Supplement: Supplementary file 9 — Predicted changes in habitat suitability from present to the 2050s of cultivated rice Oryza sativa, considering both rainfed and irrigated rice. (TIF 4570 kb) [file 12284_2017_150_MOESM9_ESM.tif]

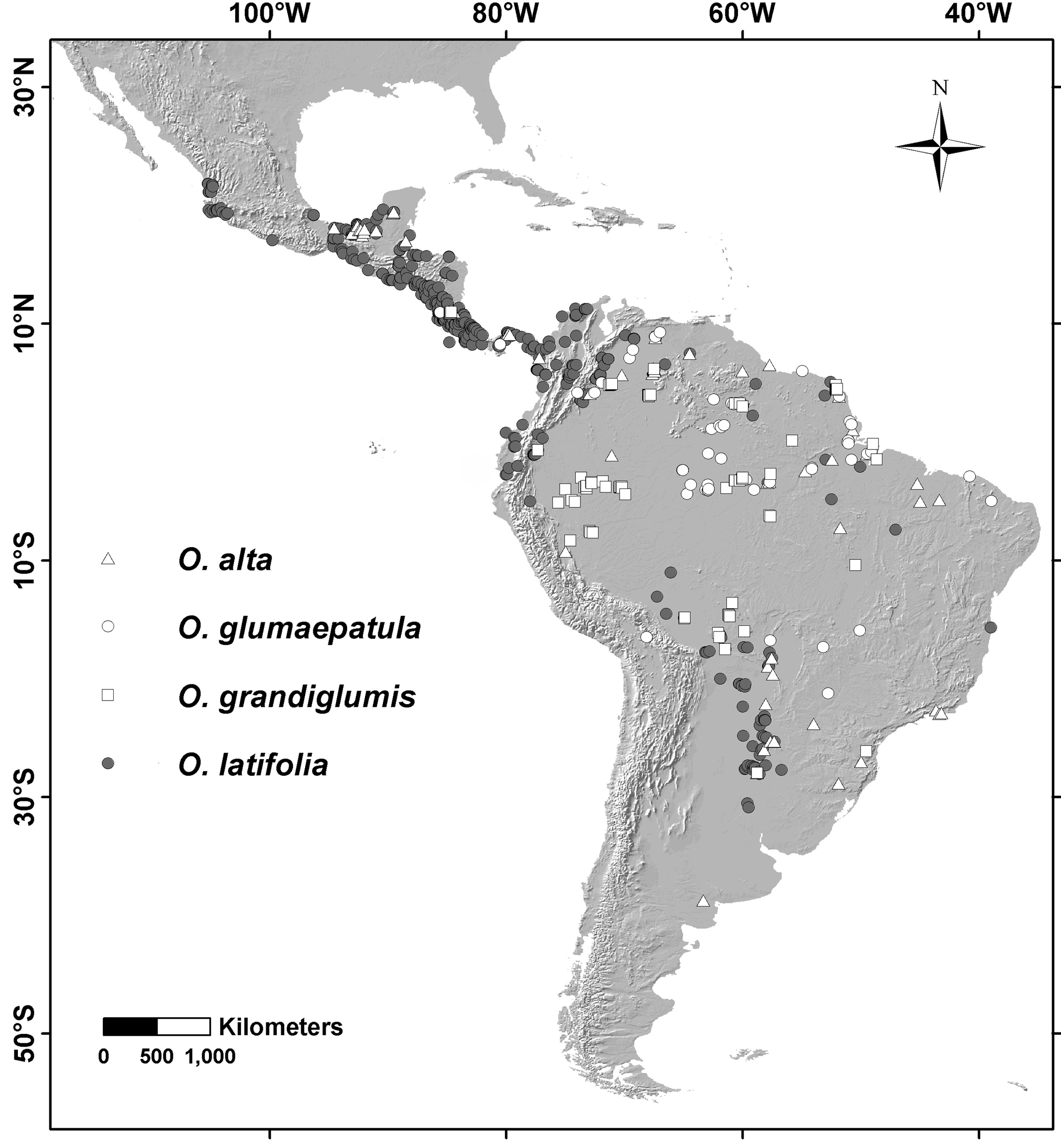

Supplement: Supplementary file 14 — Distribution of species records used in suitability model calibrations. (TIF 1695 kb) [file 12284_2017_150_MOESM14_ESM.tif]
