# Supplementary material for: Distribution, genetic diversity and potential spatiotemporal scale of alien gene flow in crop wild relatives of rice (Oryza spp.) in Colombia
Source: Rice (N Y). 2017 Apr 18;10:13. doi: 10.1186/s12284-017-0150-9 (PMC5395511; doi:10.1186/s12284-017-0150-9)
Supplement: Supplementary file 8 — Results of Mantel tests for all rice CWR between genetic and geographical distances for all individuals sampled and individuals per genetic cluster. (DOCX 14 kb) [file 12284_2017_150_MOESM8_ESM.docx]

| **Additional file 13: Table S6 Results of Mantel tests for all rice CWR between genetic and geographical distances for all individuals sampled and individuals per genetic cluster** | | | | | | |
| --- | --- | --- | --- | --- | --- | --- |
|  |  | **All individuals** | **Cluster 1** | **Cluster 2** | **Cluster 3** | **Cluster 4** |
| ***O. alta*** | **r** | 0.43 | 0.53 | 0.32 |  |  |
|  | **p** | 0.0001 | 0.046 | 0.0001 |  |  |
| ***O. grandiglumis*** | **r** | 0.99 | 0.23 | 0.66 |  |  |
|  | **p** | 0.0001 | 0.013 | 0.0001 |  |  |
| ***O. latifolia*** | **r** | 0.68 | 0.52 | -0.08 | 0.69 | 0.67 |
|  | **p** | 0.0001 | 0.0001 | 0.76 | 0.0001 | 0.0001 |
| ***O. glumaepatula*** | **r** | 0.79 | 0.77 |  |  |  |
|  | **p** | 0.0001 | 0.0001 |  |  |  |
