# Supplementary material for: Distribution, genetic diversity and potential spatiotemporal scale of alien gene flow in crop wild relatives of rice (Oryza spp.) in Colombia
Source: Rice (N Y). 2017 Apr 18;10:13. doi: 10.1186/s12284-017-0150-9 (PMC5395511; doi:10.1186/s12284-017-0150-9)
Supplement: Supplementary file 10 — Metrics obtained from genetic diversity studies of O. glumaepatula in Latin America. (DOCX 12 kb) [file 12284_2017_150_MOESM10_ESM.docx]

| **Additional file 15: Table S7 Metrics obtained from genetic diversity studies of *O. glumaepatula* in Latin America** | | | | | | | |
| --- | --- | --- | --- | --- | --- | --- | --- |
| **Metric** | **SSR** | | |  | **Isozyme** | | **Allozyme** |
|  | Present study  Colombia | Karasawa et al. 2007a  Brazil | Brondani et al. 2005  Brazil | Abreu et al. 2015  Brazil  (n=195) | Veasey et al. 2008a  Brazil | Buso et al. 1998  S. America | Akimoto et al. 1998  Brazil |
|  | (n = 23) | (n = 310) | (n =414) |  | (n = 333) | (n = 192) | (n = 1090) |
| **A** | 2.7 | 3.1 | 10.3 | 8.2 | 1-1.5 | 1-1.8 | 1.2 |
| **H_O_** | 0.141 | 0.091 | 0.027 |  | 0.005 | 0-0.025 | 0.003 |
| **H_E_** | 0.208 | 0.393 | 0.115 | 0.212-0.245 | 0.060 | 0-0.210 | 0.044 |
| **F_IS_** | 0.374 | 0.780 | 0.794 | 0.59 | 0.905 | 0.930 | 0.931 |
| **F_ST_** | 0.655 | 0.491 | 0.847 | 0.59 | 0.770 | 0.310 | 0.346 |

A = number of alleles per locus, H_E_ = expected heterozygosity, H_O_ = observed heterozygosity, F_IS_= inbreeding coefficient, F_ST=_ proportion of the total genetic variance contained in a subpopulation (the S subscript) relative to the total genetic variance
