# Supplementary material for: Distribution, genetic diversity and potential spatiotemporal scale of alien gene flow in crop wild relatives of rice (Oryza spp.) in Colombia
Source: Rice (N Y). 2017 Apr 18;10:13. doi: 10.1186/s12284-017-0150-9 (PMC5395511; doi:10.1186/s12284-017-0150-9)
Supplement: Supplementary file 11 — Details of panicles (top), ligules and auricles (bottom) of (A) Oryza latifolia with flower size smaller than 7 mm; (B) Oryza grandiglumis with approximately equal-sized sterile and fertile lemmas; and (C) Oryza alta with flower size larger than 7 mm (photo credits: Oscar Andrés Estrada). (DOCX 180 kb) [file 12284_2017_150_MOESM11_ESM.docx]

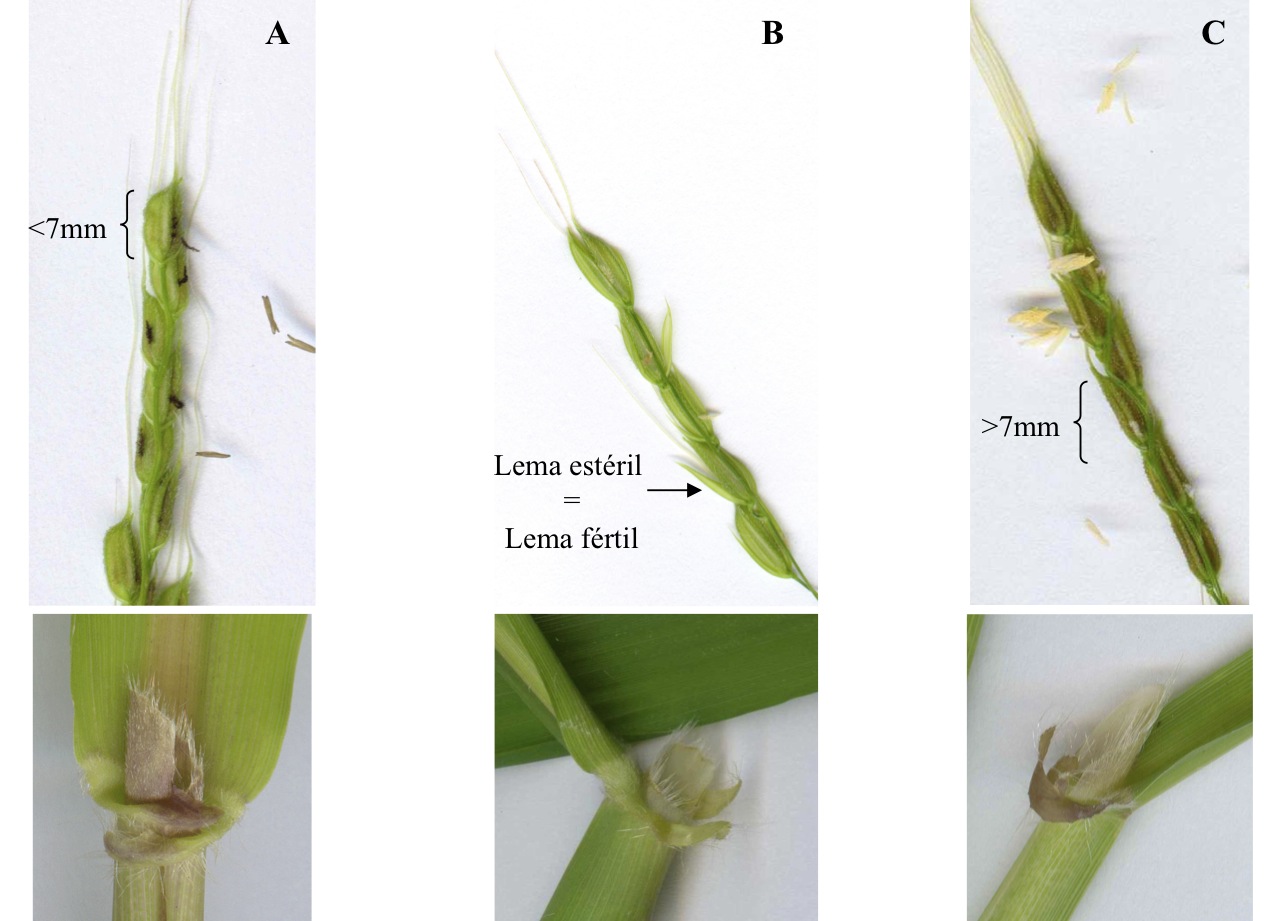


**Additional file 1: Figure S1. Details of panicles (top), ligules and auricles (bottom) of (A) *Oryza latifolia* with flower size smaller than 7 mm; (B) *Oryza grandiglumis* with approximately equal-sized sterile and fertile lemmas; and (C) *Oryza alta* with flower size larger than 7 mm.**
