# Supplementary material for: Distribution, genetic diversity and potential spatiotemporal scale of alien gene flow in crop wild relatives of rice (Oryza spp.) in Colombia
Source: Rice (N Y). 2017 Apr 18;10:13. doi: 10.1186/s12284-017-0150-9 (PMC5395511; doi:10.1186/s12284-017-0150-9)
Supplement: Supplementary file 15 — Suitability model statistics and metrics. (DOCX 17 kb) [file 12284_2017_150_MOESM15_ESM.docx]

| **Additional file 5: Table S3 Suitability model statistics** | | | |
| --- | --- | --- | --- |
|  |  | **Model calibration for projection to past (**2.5min ~4.625km at equator) | **Model calibration for projection to future (**30s ~1km at equator) |
| ***Oryza alta*** | | | |
| **Presence cells** (134 sites) | | 74 | 84 |
| **Explanatory variables** | | Bios 2, 3, 8, 13, 14, 15, 18, 19 | Bios 2, 4, 7, 8, 15, 19, elevation, aspect, slope, terrain roughness index, direction water flow, soil |
| **Best ensemble** | | (cAUC) MAXENT, FDA | (AUC) MAXENT, GBM, RF, GLM, GLMSTEP, GAM, GAMSTEP, MGCV, EARTH, RPART, NNET, FDA, SVME, BIOCLIM |
| **AUC** |  | 0.95 | 0.99 |
| **threshold** | | 0.11 | 0.05 |
| ***Oryza glumaepatula*** | | | |
| **Presence cells** (98 sites) | | 69 | 75 |
| **Explanatory variables** | | Bios 2, 3, 4, 5, 15, 18, 19 | Bios 2, 4, 7, 8, 15, 19, aspect, slope, terrain roughness index, direction water flow, soil |
| **Best ensemble** | | (cAUC) MAXENT, GAMSTEP, FDA | (AUC) MAXENT, GBM, RF, GLM, GLMSTEP, GAM, GAMSTEP, MGCV, EARTH, FDA, BIOCLIM |
| **AUC** |  | 0.93 | 0.99 |
| **threshold** | | 0.12 | 0.09 |
| ***Oryza grandiglumis*** | | | |
| **Presence cells** (130 sites) | | 78 | 89 |
| **Explanatory variables** | | Bios 2, 3, 5, 13, 14, 15, 18, 19 | Bios 2, 4, 7, 8, 13, 14, elevation, aspect, slope, terrain roughness index, direction water flow, soil |
| **Ensemble** | | (cAUC no model better) MAXENT | (cAUC) MAXENT, GBM, RF, GAM, MGCV, NNET |
| **AUC** | | 0.96 | 0.99 |
| **threshold** | | 0.27 | 0.12 |
| ***Oryza latifolia*** | | | |
| **Presence cells** (833 sites) | | 427 | 519 |
| **Explanatory variables** | | Bios 2, 3, 8, 13, 14, 15, 18, 19 | Bios 2, 4, 7, 8, 15, 18, 19, aspect, slope, terrain roughness index, direction water flow, soil |
| **Ensemble** | | (cAUC) MAXENT, RF | (cAUC) RF, RPART, BIOCLIM |
| **AUC** | | 1 | 1 |
| **threshold** | | 0.20 | 0.15 |
| ***O sativa* (irrigated)** | | | |
| **Presence cells** (420 sites) | |  | 349 |
| **Explanatory variables** | |  | Bios 2, 3, 4, 7, 8, 14, 15, 18, 19, aspect, slope, terrain roughness index, direction water flow, soil |
| **Ensemble** | |  | (cAUC) MAXENT, GBM, RF, GLM, GLMSTEP, GAM, GAMSTEP, MGCV, EARTH, RPART, NNET, FDA, SVME, BIOCLIM |
| **AUC** | |  | 0.97 |
| **threshold** | |  | 0.09 |
| ***O sativa* (rainfed)** | | | |
| **Presence cells** (299 sites) | |  | 236 |
| **Explanatory variables** | |  | Bios 2, 4, 7, 8, 15, 18, 19, aspect, slope, terrain roughness index, direction water flow, soil |
| **Ensemble** | |  | (cAUC) MAXENT, GBM, RF, GLM, GLMSTEP, GAM, GAMSTEP, MGCV, EARTH, RPART, NNET, FDA, SVME, BIOCLIM |
| **AUC** | |  | 0.97 |
| **threshold** | |  | 0.06 |
